# Independent Recruitment of Different Types of Phospholipases A2 to the Venoms of Caenophidian Snakes: The Rise of PLA_2_-IIE within Pseudoboini (Dipsadidae)

**DOI:** 10.1093/molbev/msad147

**Published:** 2023-06-23

**Authors:** Juan David Bayona-Serrano, Felipe Gobi Grazziotin, David Salazar-Valenzuela, Richard H Valente, Pedro Gabriel Nachtigall, Monica Colombini, Ana Moura-da-Silva, Inacio Loiola Meirelles Junqueira-de-Azevedo

**Affiliations:** Laboratório de Toxinologia Aplicada (LETA), Instituto Butantan, São Paulo, Brazil; Laboratório de Coleções Zoológicas (LECZ), Instituto Butantan, São Paulo, Brazil; Centro de Investigación de la Biodiversidad y Cambio Climático (BioCamb) e Ingeniería en Biodiversidad y Recursos Genéticos, Facultad de Ciencias del Medio Ambiente, Universidad Indoamérica, Quito, Ecuador; Laboratório de Toxinologia, Instituto Oswaldo Cruz, Rio de Janeiro, Brazil; Laboratório de Toxinologia Aplicada (LETA), Instituto Butantan, São Paulo, Brazil; Laboratório de Imunopatologia, Instituto Butantan, São Paulo, Brazil; Laboratório de Imunopatologia, Instituto Butantan, São Paulo, Brazil; Laboratório de Toxinologia Aplicada (LETA), Instituto Butantan, São Paulo, Brazil; Center of Toxins, Immune-Response and Cell Signaling (CeTICS), São Paulo, Brazil

**Keywords:** phospholipases A_2_, protein family evolution, gene co-option, snake venom, Dipsadidae

## Abstract

Snake venoms harbor a wide and diverse array of enzymatic and nonenzymatic toxic components, allowing them to exert myriad effects on their prey. However, they appear to trend toward a few optimal compositional scaffolds, dominated by four major toxin classes: SVMPs, SVSPs, 3FTxs, and PLA_2_s. Nevertheless, the latter appears to be restricted to vipers and elapids, as it has never been reported as a major venom component in rear-fanged species. Here, by investigating the original transcriptomes from 19 species distributed in eight genera from the Pseudoboini tribe (Dipsadidae: Xenodontinae) and screening among seven additional tribes of Dipsadidae and three additional families of advanced snakes, we discovered that a novel type of venom PLA_2_, resembling a PLA_2_-IIE, has been recruited to the venom of some species of the Pseudoboini tribe, where it is a major component. Proteomic and functional analyses of these venoms further indicate that these PLA_2_s play a relevant role in the venoms from this tribe. Moreover, we reconstructed the phylogeny of PLA_2_s across different snake groups and show that different types of these toxins have been recruited in at least five independent events in caenophidian snakes. Additionally, we present the first compositional profiling of Pseudoboini venoms. Our results demonstrate how relevant phenotypic traits are convergently recruited by different means and from homologous and nonhomologous genes in phylogenetically and ecologically divergent snake groups, possibly optimizing venom composition to overcome diverse adaptative landscapes.

## Introduction

Venomous animals and their toxins have been increasingly scrutinized by researchers from around the world in the last four decades (Fox and Serrano 2007; Fry 2015; Zhang 2015; Mackessy 2021). Studies aiming to understand and alleviate the epidemiological phenomenon of human envenomation by these animals were the main drivers of the toxinological sciences throughout most of human history (Prado-Franceschi and Hyslop 2002; Otero-Patiño 2009; Junqueira-de-Azevedo et al. 2016; Mackessy 2021; Sevilla-Sánchez et al. 2021). Among the great diversity of vertebrate and invertebrate venomous animals, snakes cause higher proportions of human accidents and deaths worldwide (Prado-Franceschi and Hyslop 2002; Chippaux 2015; Williams et al. 2019). Therefore, venoms from medically relevant snake species belonging to the Viperidae and Elapidae families, which are characterized by their front-fanged dentitions, are among the better-known animal secretions (Fry 2015; Mackessy 2021). However, most snake diversity lies elsewhere, mainly within the family Dipsadidae (superfamily: Colubroidea), which contains venom-producing species that have been historically neglected in toxinological studies due to their low medical relevance (Junqueira-de-Azevedo et al. 2016; Uetz et al. 2019; Zaher et al. 2019). These snakes exhibit a diverse array of ecological and morphological traits, which might be mirrored by equally varied venoms (Henderson 1982; de Oliveira et al. 2008; Barbo et al. 2011; Weinstein et al. 2011; Gaiarsa et al. 2013; Giraudo et al. 2014).

Recently, there has been an increased number of works that address venom-related questions for the abovementioned species that reveal highly complex venom compositions, which resemble the characteristics observed in vipers and elapids. Moreover, these works have revealed a series of new, poorly characterized venom components that appear to be found only in this group of snakes (de Oliveira et al. 2008; Weinstein et al. 2011; Campos et al. 2016; Junqueira-de-Azevedo et al. 2016; Modahl et al. 2018; Bayona-Serrano et al. 2020; Mackessy 2021). However, there is still a lack any kind of compositional or functional information about the venoms of most dipsadid genera (Junqueira-de-Azevedo et al. 2016; Barua and Mikheyev 2019; Bayona-Serrano et al. 2020). The species addressed thus far have indicated that proteolytic enzymes (e.g., zinc-dependent metalloproteinases), followed by cysteine-rich secretory proteins (CRiSPs) and C-type lectins (CTLs), tend to dominate in their venoms (Ching et al. 2012; Campos et al. 2016; Junqueira-de-Azevedo et al. 2016; Bayona-Serrano et al. 2020). Remarkably, phospholipases A_2_ (PLA_2_s), a common denominator in the venoms of front-fanged snakes and one of the major effectors of their toxic actions (e.g., myotoxicity, myonecrosis, lipid membrane damage, neurotoxicity, and prey immobilization), have not been assertively associated with venom features in any dipsadid (Junqueira-de-Azevedo et al. 2016; Barua and Mikheyev 2019).

PLA_2_s catalyze the hydrolysis of glycerophospholipids at the *sn-2* position, producing free fatty acids and lysophospholipids (Six and Dennis 2000; Huang et al. 2015). They are ubiquitous in vertebrates, where they fulfill several physiological roles and are divided into three main categories: secretory, cytosolic, and Ca^2+^-independent PLA_2_s, depending on their cellular location and catalytic mechanism (Six and Dennis 2000). Snake venom PLA_2_s belong to the secretory type, which has been classically divided into 11 different groups based on their primary structures and the tissues in which they are most commonly expressed (Six and Dennis 2000). Venom PLA_2_s have been commonly reported in two front-fanged snake families: the Elapidae family possesses secretory PLA_2_s from group I and the Viperidae family produces group IIA PLA_2_s in its venoms (Ogawa et al. 1995; Jeyaseelan et al. 2000; Huang et al. 2015; Dowell et al. 2016). These enzymes are generally associated with some of the most severe symptoms observed in accidents involving front-fanged snakes that harbor them as a major toxin class (Gutiérrez et al. 2009; Tsai 2016; Pla et al. 2018; Tasoulis et al. 2020; Mackessy 2021). These enzymes have undergone accelerated evolution and genetic expansion in these front-fanged snakes families, where they represent a multigene family with several paralogs (Jeyaseelan et al. 2000; Yamaguchi et al. 2014; Dowell et al. 2016; Suranse et al. 2022).

The contribution of PLA_2_s to the venoms of rear-fanged snakes is less evident, and their activity, abundance, structural diversity, and evolution might be underestimated (Huang and Mackessy 2004; Fry et al. 2012; Fry 2015; Junqueira-de-Azevedo et al. 2016; Pla et al. 2017; Torres-Bonilla et al. 2018; Mackessy et al. 2020). In 2004, a protein showing PLA_2_ catalytic activity and high similarity at its *N*-terminus to PLA_2_-IA from sea snakes was isolated from the venom of the colubrid, *Trimorphodon lambda* (Huang and Mackessy 2004). However, a few other rear-fanged species were shown to have a different type of PLA_2_ in their venoms, PLA_2_-IIE, which commonly occurs in the human and mouse brain/heart/uterus and serves physiological functions (Six and Dennis 2000; Suzuki et al. 2000; Mackessy 2021). Transcripts for PLA_2_-IIE have been detected at low levels in the venom glands of several snakes, although they never appear to be as preponderant as their I or IIA counterparts found in elapids or vipers, respectively (Fry et al. 2012; Yamaguchi et al. 2014; Pla et al. 2017). *Dispholidus typus* and *Oxyrhopus guibei*, species from two completely different families (i.e.., Colubridae and Dipsadidae, respectively), have the highest expression levels of PLA_2_-IIE transcripts among rear-fanged snakes reported thus far (Junqueira-de-Azevedo et al. 2016; Pla et al. 2017). The former is a colubrid that is notorious for its potent venom, being involved in human casualties, including the renowned case of Karl Patterson Schmidt (Pla et al. 2017). On the other hand, *O. guibei*, a dipsadid belonging to the Pseudoboini tribe, is a docile species with a scarce record of human accidents. In 2018, Torres-Bonilla et al. studied the enzymatic actions of the venom of the pseudoboine *Pseudoboa neuwiedii* and found that it had PLA_2_ activity levels similar to those observed in viper species. Subsequent proteomic analyses of the venom of *P. neuwiedii* identified peptides belonging to the PLA_2_s of group II (Torres-Bonilla et al. 2018). This finding, along with a previous report of PLA_2_-IIE transcripts being expressed in the venom gland of *O. guibei*, hinted that PLA_2_s might be a relevant venom component in the tribe Pseudoboini.

In this work, we elucidated the occurrence of PLA_2_s in the venoms of the Dipsadidae family by scrutinizing the venoms and venom glands of the Pseudoboini tribe and additional outgroups through transcriptomic, proteomic, and functional approaches. We reconstructed the evolutionary history of PLA_2_-IIE in snakes and discuss the possible recruitment and duplication events that turned this family of proteins into a major player in dozens of rear-fanged species. Moreover, we contrast our findings with previously reported PLA_2_s from other snake families and infer that multiple recruitment events have shaped the dispersion of these toxins across caenophidian snakes. Additionally, the data regarding other toxins of the Pseudoboini tribe represent a substantial addition to the poor knowledge of the venom compositions of rear-fanged snakes and bring new research opportunities for the exploration of colubroid venoms.

## Results

### Venom Compositional Profile of the Pseudoboini Tribe: A PLA_2_-IIE–Rich Group of Snakes

**Fig. 1.**
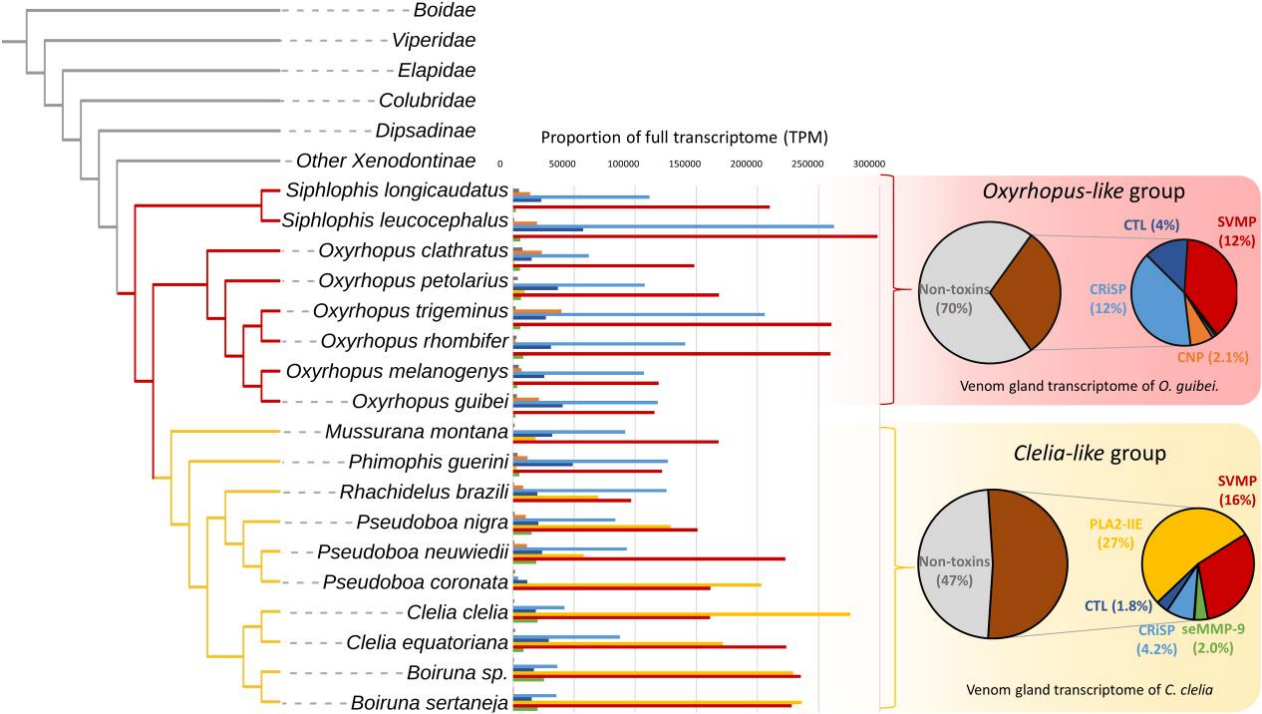
Compositional profiles of venom-related transcripts in the Pseudoboini tribe. The phylogenetic tree was adapted from ZAHER et al. 2019 for general relationships outside of the tribe and was derived from our phylogenetic analyses for relationships within the tribe (supplementary fig. S1, Supplementary Material online). Bars represent the average amount of each annotated toxin type in the species. Pie plots to the right are from a representative species for each group. Note the elevated amount of PLA_2_ present in the Clelia-like group.

To establish the phylogenetic relationships of species within the Pseudoboini tribe, which is associated with their venom profiles, we used our assembled transcriptomic data to build a data set of 2,161 conserved loci and used them to reconstruct the phylogenetic relationships of the tribe (supplementary fig. S1, Supplementary Material online). The full mitochondrial genomes of the individuals were also recovered using the MITGARD approach and used to reconstruct the phylogenetic relationships of the tribe (supplementary fig. S2, Supplementary Material online) (Nachtigall, Grazziotin, et al. 2021). Both trees showed similar relationships, but we adopted the tree based on the conserved loci to draw the illustrative tree shown in figure 1 due to its higher node support values.

Venom gland transcriptome (VGT) annotation of species from this tribe uncovered a wide array of toxin classes, both enzymatic and nonenzymatic, with a varying number of putative paralogs and expression levels (fig. 1 and supplementary table S1, Supplementary Material online). Snake venom metalloproteinases from the *P*-III subtype (SVMP P-III) were a dominant component in all species of the tribe. Despite this protease dominance, we observed great compositional variations, especially regarding CRiSPs and PLA_2_s. Based on its toxin expression profiles, the tribe could be divided into two main groups: the *Oxyrhopus-like* group and the *Clelia-like* group (fig. 1).

The *Oxyrhopus-like* group contains species from the genera *Oxyrhopus* and *Siphlophis* that possess VGTs dominated by SVMP-PIII and CRiSPs, with lower expressions of CTLs, natriuretic peptides (CNP), snake endogenous matrix metalloproteinases (seMMP-9), and PLA_2_-IIE. On the other hand, the *Clelia-like* group contains the genera *Mussurana*, *Phimophis*, *Rhachidelus*, *Pseudoboa*, *Boiruna*, and *Clelia*. These genera form a monophyletic group (supplementary fig. S1, Supplementary Material online) and showed similar expression levels of minor toxins but an overall higher proportion of PLA_2_-IIE, which was the dominant toxin class in some species. Within the *Clelia-like* group, the genera *Pseudoboa*, *Boiruna*, and *Clelia* showed the highest expression levels of PLA_2_-IIE and a PCA confirmed that species belonging to these genera cluster closer together and away from other species of the tribe and are more compositionally related (supplementary fig. S3, Supplementary Material online).

**Fig. 2.**
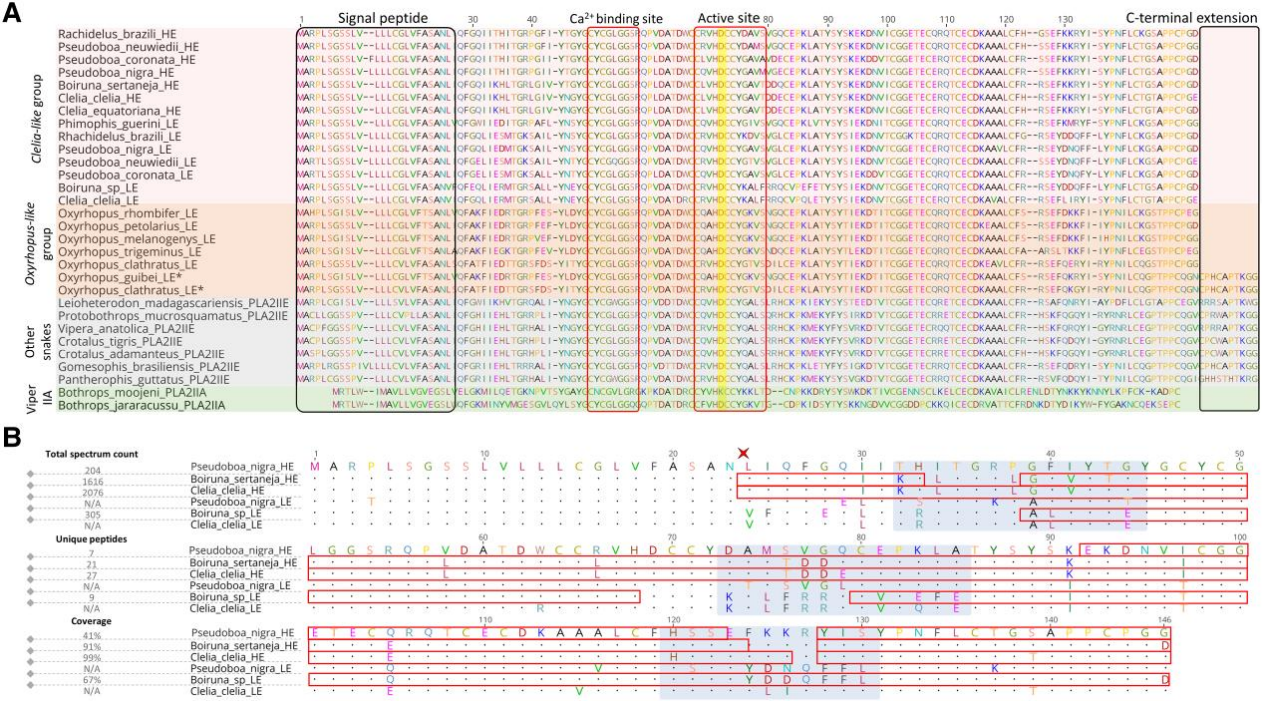
(*A*) Multiple sequence alignment of PLA_2_-IIEs from Pseudoboini and other snakes. Structural features are indicated by closed boxes. The aspartate residue, typical in catalytically active PLA2*s*, is highlighted in yellow. The asterisks indicate Pseudoboini PLA2-IIE with the C-terminal extension. The accession numbers for the sequences from other snakes are gi_698375631, gi_384110785, XM_015820366, MN831292, XM_039367457, gi_1147529007, gi_25140376, and KX211996. (*B*) Protein sequence alignment of the highly and lowly expressed transcripts from representative species of the Clelia-like group. Dots indicate conserved amino acids between aligned sequences. Peptides identified by proteomic analyses are highlighted with closed boxes. Heterogeneous regions found across the different proteins are indicated with light-blue shading. The end of the signal peptide is marked by a red cross above residue 24. Total spectrum counts, unique peptides, and coverages of mature proteins are indicated for identified proteins.

Most species from the *Oxyrhopus-like* group retained only one PLA_2_-IIE transcript, the exception being *Oxyrhopus clathratus*, which retained two PLA_2_-IIE transcripts after curation of their VGT (supplementary table S1, Supplementary Material online). On the other hand, all species in the *Clelia-like* group possess two different PLA_2_-IIE transcripts that show radically different expression levels, one very highly expressed and the other lowly expressed. When looking at the primary amino acid structure of the PLA_2_-IIE-derived proteins retained for species of the tribe, we determined that all PLA_2_-IIEs from the *Clelia-like* group encoded shorter proteins, which lack a portion of the C-terminal that is present in some of the *Oxyrhopus* group-derived proteins and is the typical structure in endogenous PLA_2_-IIEs from other snake groups (fig. 2*A*).

However, *O. clathratus* had both the long and short forms. Multiple sequence alignments of full-length PLA_2_-IIE transcripts revealed that this shorter C-terminus is caused by deletions of 30 and 21 bp in Pseudoboini and in the colubrid *D. typus*, respectively, while all other PLA_2_-IIEs from the analyzed snakes possess a longer C-terminus (supplementary fig. S4, Supplementary Material online). Moreover, when comparing the primary structures from representative sequences of the highly and weakly expressed transcripts from the genera *Pseudoboa*, *Boiruna*, and *Clelia* (*Clelia-like* group), we determined that they possess three different heterogeneous portions between them, even though their signal peptides and active sites were similar (fig. 2*B*).

**Fig. 3.**
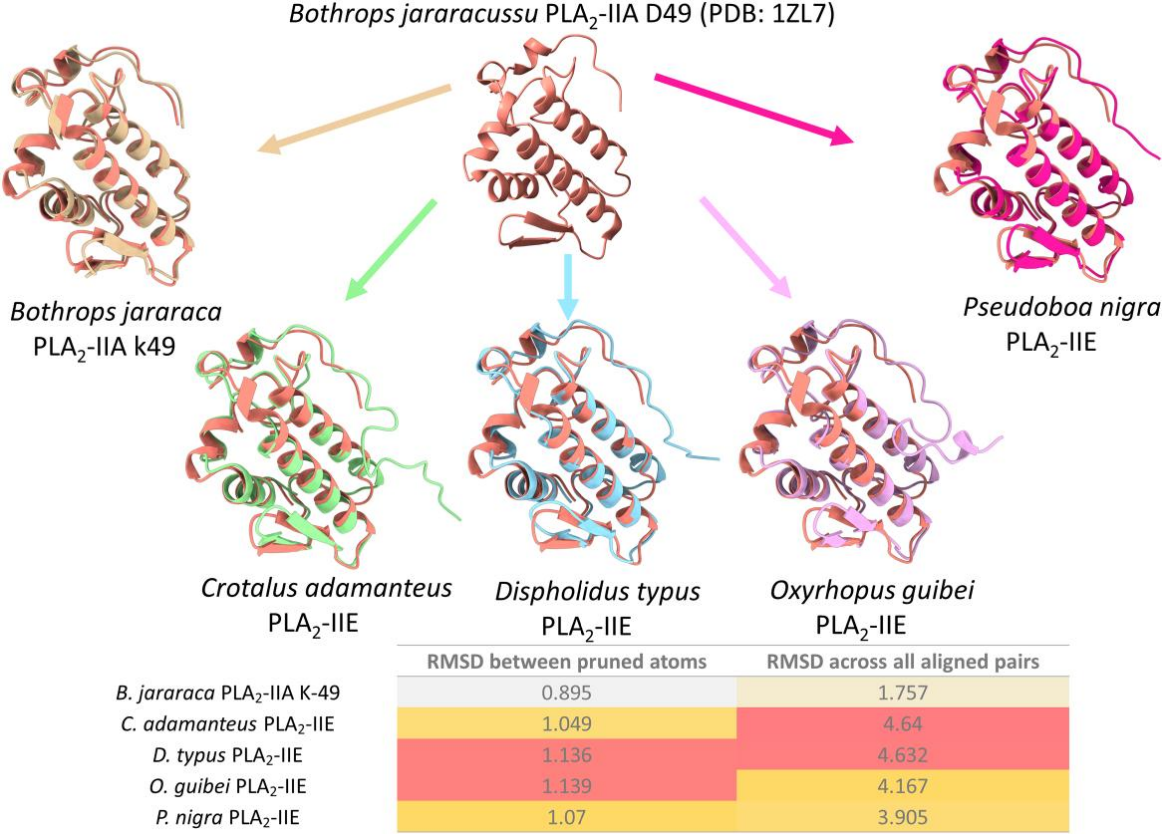
Three-dimensional alignments of a *B. jararacussu* PLA_2_-IIA with different PLA_2_ forms from vipers and colubrid species. The table below displays the RMSD scores for each pairwise alignment. Yellow tones indicate better (lower) scores.

Proteomic analyses not only confirmed the occurrence of PLA_2_-IIE in the venoms of *Pseudoboa nigra*, *Boiruna sertaneja*, and *Clelia equatoriana* but also indicated that it was a major constituent of these venoms (fig. 2*B* and supplementary fig. S5, Supplementary Material online). Moreover, the predicted protein from the highly expressed transcript in the venom glands was detected in all three species, harboring a higher proportion of mass spectra than the protein from the weakly expressed transcript, even when both were identified as occurring in *B. sertaneja* (supplementary table S2, Supplementary Material online). In *Clelia clelia* and *P. nigra*, only the highly expressed form was detected in the proteome. Other major venom components reported in the VGTs of the tribe, such as SVMPs, CRiSPs, and seMMP-9, were also confirmed to be present in the venom of these three species (fig. 3 and supplementary table S2, Supplementary Material online). Moreover, the abundances of identified venom toxins estimated in Scaffold 5 followed the same compositional trends that we observed in VGTs, with SVMPs and PLA_2_-IIE being dominant toxins in *C. equatoriana* and *B. sertaneja* (supplementary fig. S5, Supplementary Material Online).

The 3D structures of a highly expressed PLA_2_-IIE from *P. nigra*, used as a representative from the *Clelia-like* group; the longer PLA_2_-IIE from *O. guibei*, a PLA_2_-IIE from *D. typus*; a PLA_2_-IIE from *Crotalus adamanteus*; and a noncatalytic PLA_2_-IIA from *Bothrops jararaca*, were predicted with RoseTTAFold (Hiranuma et al. 2021). Comparisons between these structures and the 3D crystal structure of a catalytically active PLA_2_-IIA from *Bothrops jararacussu* revealed that some of the shorter PLA_2_-IIE forms obtained from the *Clelia-like* group have better RMSD scores across all aligned pairs than the PLA_2_-IIE forms obtained from other viper species (fig. 3).

### PLA_2_-IIEs Inside and Outside of the Pseudoboini Tribe

**Fig. 4.**
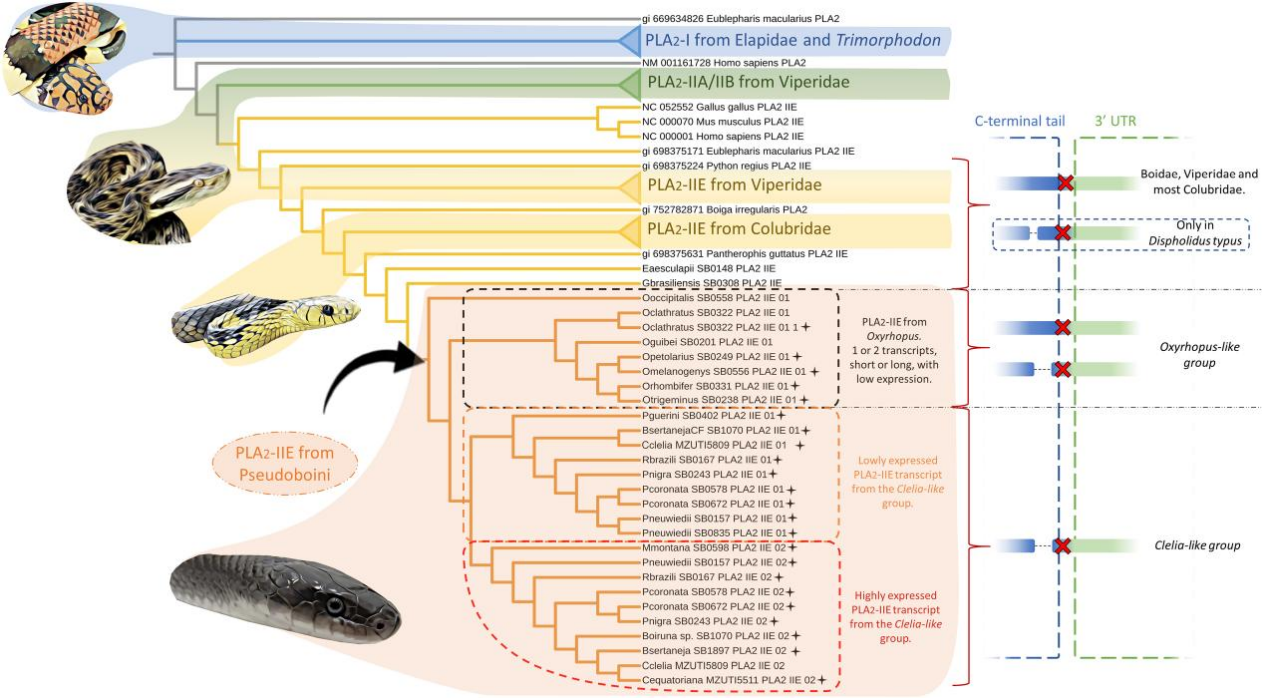
Phylogenetic reconstruction of the assembled PLA_2_-IIE from Pseudoboini with PLA_2_*s* from other snakes using 1,000 ultrafast bootstrap replicates. Assembled PLA_2_*s* from Pseudoboini cluster within the IIE subgroup, which is the sister group of PLA_2_*s*-IIA from vipers. Black crosses within the Pseudoboini tribe indicate sequences without the C-terminal extension commonly seen in endogenous PLA_2_-IIEs from other snakes. A schematic representation of multiple sequence alignments of full transcripts of PLA_2_-IIEs across sampled species exhibiting the variation of the C-terminal arrangement is shown to the right. The C-terminal region of the coding sequence is highlighted in blue. The 3′UTR of the full transcript is highlighted in green. *D. typus* (Colubridae), some *Oxyrhopus*, and all species from the Clelia-like group present a deletion before the stop codon, as indicated by red crosses.

The elevated expression levels of PLA_2_-IIE found within the Pseudoboini tribe led us to wonder if these were a unique characteristic of this group within dipsadids or if PLA_2_s were also dominant in other tribes. We gathered previously generated snake venom transcriptomic data for representative species of seven additional tribes of Dipsadidae and for the available species of Colubridae, Elapidae, and Viperidae and screened for PLA_2_-IIE–like sequences, as we performed in a previous work (Bayona-Serrano et al. 2020). The analysis indicated that the elevated expression levels of PLA_2_-IIE in venom glands are likely exclusive to the Pseudoboini tribe within Dipsadidae (supplementary fig. S6, Supplementary Material online), suggesting that, when present in other groups, the PLA_2_-IIE transcript may correspond to the endophysiological protein. A phylogenetic tree reconstruction performed using PLA_2_ sequences from elapids, vipers, colubroids, and other vertebrates revealed that the PLA_2_s recovered from Pseudoboini species are nested within the type IIE of PLA_2_s from other vertebrates. PLA_2_-IIE is the sister group of PLA_2_-IIA found on viper venoms (fig. 4 and supplementary figs. S7, S8, S9, and S10, Supplementary Material online). Moreover, PLA_2_-IIEs recovered from the *Oxyrhopus* group separate themselves from those of the *Clelia-like* group. Interestingly, within the *Clelia-like* group, the highly and weakly expressed transcripts formed separate independent groups. An orthology analysis performed with OrthoFinder clustered all assembled PLA_2_-IIEs from Pseudoboini within a single orthogroup, which was probably due to their overall sequence similarity.

### Enzymatic Assays

**Fig. 5.**
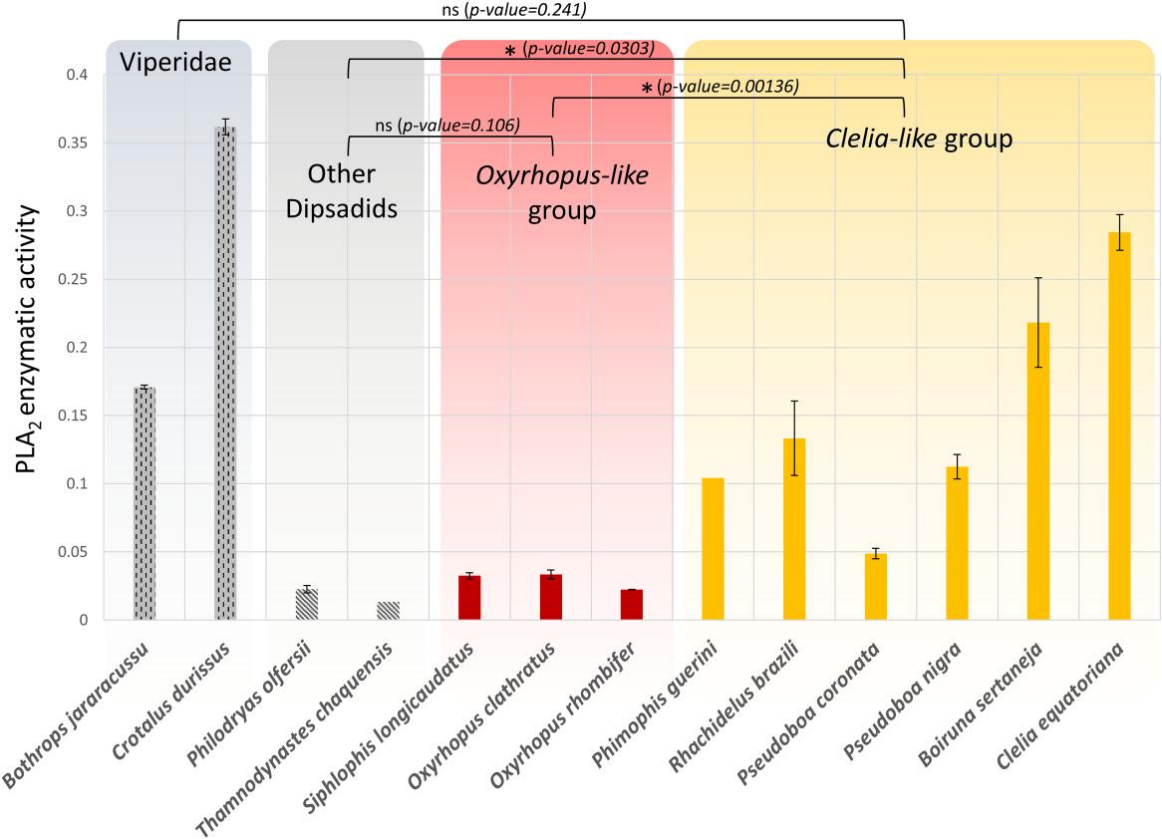
PLA_2_ enzymatic activities measured for Pseudoboini venoms. Each color represents a different group of samples. Asterisks indicate significant differences between groups. SEM is plotted for each bar.

Some Pseudoboini venoms tested for PLA_2_ activity showed activities comparable to those of viper venoms (fig. 5). Comparisons were made among four different groups: venoms from viper species known to possess PLA_2_ activity, venoms from dipsadids shown to be devoid of PLA_2_s, venoms from species from the *Oxyrhopus* group, and venoms from species from the *Clelia-like* group. We observed significant differences between the PLA_2_ activities found in species from the *Clelia-like* group and species from the *Oxyrhopus* group. The latter were statistically equal to venom from other dipsadids. For the comparison between activity levels from viper venoms and venoms from the *Clelia-like* group, we were unable to find statistically significant differences, although crude venoms from *C. Clelia* and *B. sertaneja* presented higher enzymatic activity values than the crude venom of *B. jararacussu*, which is known to be one of the most PLA_2_-rich species of snake (Freitas-De-sousa et al. 2020) (fig. 5).

## Discussion

The phylogenetic analyses allowed us to reconstruct a robust topology for the sampled species of the Pseudoboini tribe and take advantage of the multigene approach that high-throughput transcriptomics generate. Our results allowed us to resolve some internal relationships within the tribe, which clustered the genus *Rhachidelus* along with the monophyletic group formed by *Pseudoboa*, *Clelia*, and *Boiruna* (Zaher et al. 2019) (supplementary figs. S1 and S2, Supplementary Material online).

The venoms from the Pseudoboini tribe showed the same protease-rich profiles observed in most dipsadids (Junqueira-de-Azevedo et al. 2016; Bayona-Serrano et al. 2020). SVMPs remain a major toxin class in the venoms of these snakes, and elevated levels of CTLs, CRiSPs, and seMMP-9 point to them as the usual players in Dipsadidae venoms. However, we noticed unusually high proportions of PLA_2_s in this tribe, both in their VGTs and proteomes. The PLA_2_ amounts were particularly high in the *Clelia-like* group, in which the PLA_2_-IIE expression levels ranged from ∼1% to ∼27% of whole transcriptomes (fig. 1 and supplementary table S1, Supplementary Material online). These high expression levels were found to be exclusive to the Pseudoboini tribe, at least among the species sampled in our screening (supplementary fig. S6, Supplementary Material online). All other analyzed tribes within the family had TPM values near zero for PLA_2_-like contigs. Regarding other snake families (e.g., Colubridae, Elapidae, and Viperidae) that were screened, we also found almost null expressions of PLA_2_-IIE–like contigs. However, previous works have reported PLA_2_s from both type IA and IIE as venom components in some species of the Colubridae family (Huang and Mackessy 2004; Fry et al. 2012; Pla et al. 2017; Mackessy et al. 2020). This indicates that there might be other PLA_2_s hidden in specific snake groups. An in-depth sampling of venom-producing colubroids is needed to truly determine their occurrence across all advanced snake clades.

Functional analyses of Pseudoboini venoms revealed high PLA_2_ activities, which were comparable to those of viper venoms, in species from the *Clelia-like* group (fig. 5). These findings, along with the fact that a highly expressed PLA_2_-IIE was found in the analyzed venom proteomes of the group, suggest that this form is responsible for the catalytic PLA_2_ activity observed. Previous works had already reported high PLA_2_ activity in the venom of *P. neuwiedii* (Torres-Bonilla et al. 2017, 2018). However, proteomic analyses of the venom of that species identified an ∼14–15-kDa SDS–PAGE band as PLA_2_-IIA. In that work, the identified spectra were searched against the UniProtKB/Swiss-Prot database, which found several peptides that matched a myotoxic noncatalytic PLA_2_-IIA from *Bothrops moojeni*. However, all PLA_2_s we recovered for the tribe, including *P. neuwiedii*, were catalytically active and belonged to the IIE subgroup of PLA_2_s. Therefore, we attribute the previous identification of PLA_2_-IIA in the venom of *P. neuwiedii* to the general sequence similarity between PLA_2_-IIA and PLA_2_-IIE and to the lack of representative PLA_2_-IIE from Pseudoboini in the databases used in that work (Yamaguchi et al. 2014), which hindered the correct identification of the PLA_2_ subtype. It is worth highlighting the considerable PLA_2_ activity that we measured for *Phimophis guerini*, since this species had the lowest proportion of PLA_2_-IIE in its VGT within the *Clelia-like* group (fig. 1). Increased sampling within the genus is needed to confirm whether a highly expressed PLA_2_-IIE transcript occurs within its VGTs or if the PLA_2_ activity observed is mediated by other means. Species from the *Oxyrhopus-like* group showed no significant differences from the venoms obtained from dipsadids known to be devoid of PLA_2_s, in agreement with the low PLA_2_-IIE expressions we found in their transcriptomes.

PLA_2_ enzymes have been predominantly reported as venom components of front-fanged snakes from the Viperidae family, harboring PLA_2_-IIA, and the Elapidae family, harboring PLA_2_-IA and PLA_2_-IB (Jeyaseelan et al. 2000; Dowell et al. 2016). Interestingly, the genetic organization of the PLA_2_-II locus in vipers and other vertebrates suggests that PLA_2_s from group IID, which are the evolutionary precursor of all PLA_2_-IIA from vipers, were derived from an ancestral duplication of the PLA_2_-IIE gene followed by sequential gene duplication and diversification within Viperidae, originating venom PLA_2_-IIA toxins (Yamaguchi et al. 2014; Dowell et al. 2016; Koludarov et al. 2019; Suranse et al. 2022). Moreover, genomic data have revealed the presence of exonic debris from the PLA_2_-IIE gene spread downstream from the PLA_2_-IIE gene in vipers, indicating plausible duplication and pseudogenization events (Dowell et al. 2016; Koludarov et al. 2019). To determine whether these duplications occurred before the diversification of vipers, we analyzed the available genomes of the colubrids *Thamnophis sirtalis* (NCBI accession number NW_013659820.1) and *Pantherophis guttatus* (NCBI accession number NW_023010753.1). We did not find exonic debris for the PLA_2_-IIE gene in those species, indicating that the possible duplication of the PLA_2_-IIE gene took place after viper diversification and that it does not represent a basal trait in advanced snakes. Therefore, the finding of two types of PLA_2_-IIE transcripts showing structural and quantitative differences in some Pseudoboini species reinforces the hypothesis that the PLA_2_-IIE gene has undergone at least one duplication event within the tribe.

Gene duplication is a known trigger of accelerated evolution (Ohno 1971; True and Carroll 2002) that is observed in many venom proteins (Ogawa et al. 1995, 2005; Kini and Doley 2010; Vonk et al. 2013; Dowell et al. 2016; Lomonte et al. 2016; Tadokoro et al. 2020). The two types of PLA_2_-IIE found in Pseudoboini differ not only in their sequence substitutions but also in the small deletions on their C-terminal portions (fig. 2*A*). Previous works have noted that even though the primary structures of IIA and IIE PLA_2_s are similar, the C-terminal tails are distinct between them, with PLA_2_-IIE having a longer C-terminus (Yamaguchi et al. 2014). Interestingly, a shorter C-terminal deletion was also present in the PLA_2_-IIE contig reported for the colubrid *D. typus*, which has moderate levels of PLA_2_ expression in its venom glands (∼2.75% of whole transcriptome) (Pla et al. 2017). The C-terminal deletion, which shortens the primary structure of the PLA_2_-IIE protein, observed only in *D. typus* and in all species from the *Clelia-like* group, constitutes a convergent event in rear-fanged snake groups displaying increased expression levels of this protein type in their venom glands. The role and relevance of this deletion are still not fully understood, but it might indicate a trend toward a more compact IIA-like structural scaffold. The 3D alignments favor this trend, as the PLA_2_-IIE from the *Clelia-like* group with a shortened C-terminus showed better alignment scores toward the structure of a viper PLA_2_-IIA than the PLA_2_-IIEs from other vipers, which do not possess the C-terminal deletion (fig. 2*A* and fig. 3). However, as these 3D alignments were made with predicted structures and the Armstrong error estimate of the models, calculated by RoseTTAFold, always increased toward the C-terminal portion, it is hard to assertively link this deletion to a trend toward a more IIA-like structure.

The phylogenetic reconstruction of PLA_2_s showed that PLA_2_-IIEs from Pseudoboini form a sister clade to PLA_2_-IIAs from vipers. Within the Pseudoboini tribe, the weakly expressed PLA_2_-IIE from *O. occipitalis* possessing the longer C-terminal, was the most basal protein, resembling the PLA_2_-IIE scaffold found outside of the tribe. Within the *Clelia-like* group, PLA_2_-IIE is organized into two separate clades, one containing the highly expressed transcripts and the other containing the weakly expressed transcripts, both harboring the C-terminal deletion (fig. 4). The highly expressed form was found more consistently in the venom proteome of the analyzed genera, with more spectral counts, indicating a higher relative abundance of the protein. We also found that the two clades of sequences had three heterogeneous portions on their primary structures. The implications of these differences are not clear, but it would be expected that the different residues of the highly expressed form contribute to the overall enzymatic efficiency of the protein in the venom.

Outside Pseudoboini, PLA_2_-IIE sequences can be retrieved from many snake taxa, mostly from genome annotations or PCR products amplified from various tissues, including the venom glands (Fry et al. 2012; Yamaguchi et al. 2014). There is no strong evidence, however, of PLA_2_-IIE being a relevant venom component in other snake families, with the sole exception of the colubrid, *D. typus* (Pla et al. 2017). In this case, combined transcriptomic and proteomic analyses identified PLA_2_-IIE among the top three most abundant toxins in the venom. On the other hand, our phylogenetic analysis indicated that the peculiar PLA_2_ proteins reported in the colubrid genus, *Trimorphodon*, are not PLA_2_-IIE but belong to the PLA_2_-I type, as had been previously reported (Fry et al. 2008) (fig. 4). The evolutionary history of the PLA_2_ gene family in rear-fanged snakes appears to be rather complex, as some species possess PLA_2_-IA–like proteins (e.g., *T. lambda*), while others exhibit PLA_2_-IIE–like proteins (e.g., *D. typus* and most Pseudoboini species) (Pla et al. 2017; Mackessy et al. 2020).

The genetic scaffold of the PLA_2_-II gene cluster is highly conserved in humans, mice, birds, and snakes (Huang et al. 2015). The triplet organization of the locus, with the OTUD3 gene, followed by the PLA_2_-IIE gene and then the PLA_2_-IID cluster, is mostly maintained in these groups (Huang et al. 2015; Dowell et al. 2016; Suranse et al. 2022). A PLA_2_-IID gene is assumed to be ancestrally recruited in vipers and co-opted into a venom protein, resulting in the modern PLA_2_-IIA observed in viper venoms (Yamaguchi et al. 2014; Dowell et al. 2016; Koludarov et al. 2019). This recruitment was followed by sequential gene duplication and accelerated evolution, marked by diverse substitutions at the catalytic site, ultimately generating a noncatalytic (K49) form in some vipers (Huang et al. 2015; Dowell et al. 2016; Suranse et al. 2022). However, this genetic expansion of the PLA_2_-IID cluster, derived from the PLA_2_-IIA venom forms found in vipers, has not yet been observed in any other group of advanced snakes. On the other hand, less information is known regarding the genetic scaffolding and evolutionary history of PLA_2_-I from elapids. These PLA_2_s are structurally divided into group IB, commonly found in mammalian pancreases but also reported in the venoms of some elapid snakes (Armugam et al. 2004; Mackessy 2021), and group IA, which is found almost exclusively in elapid venoms and lacks the “pancreatic loop” characteristic of group IB (Jeyaseelan et al. 2000; Huang and Mackessy 2004; Mackessy 2021). PLA_2_-I are placed in a different genomic locus, and their phylogenetic reconstruction indicates that their diversification was genus specific and influenced by the ecology and evolutionary history of each lineage (Jeyaseelan et al. 2000). Genomic data from rear-fanged species expressing PLA_2_s in their venom are needed to reveal the genomic organization of the PLA_2_-I and PLA_2_-II gene loci and determine if they are in fact undergoing similar genetic processes as the ones observed in vipers and elapids.

**Fig. 6.**
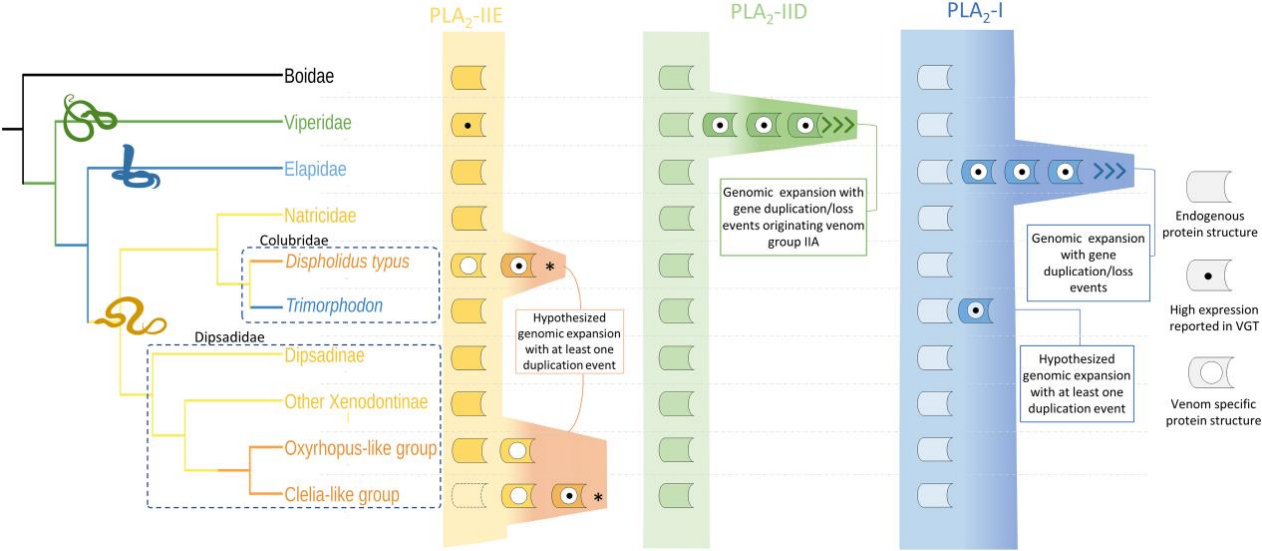
Hypothetical genomic expansion events within each PLA_2_ type among different snake families. Each colored box represents a gene. Physiological copies are assumed to be present in all snake groups. The open box shown in the Clelia-like group indicates the uncertainty of the physiological PLA_2_-IIE gene structure, as we could not find a transcript without the C-terminal deletion in the sampled species. Genomic data are needed to reveal the true arrangement of the PLA_2_-IIE gene in that group.

Based on our findings and previous literature reports, we can hypothesize at least three distinct events of recruitment and restriction of PLA_2_-like toxins into the venom glands of rear-fanged snakes (fig. 6). PLA_2_s from Group I, which are commonly found in elapid venoms, are apparently recruited to the venom glands of the genus *Trimorphodon*, which has been shown to possess PLA_2_-IA–like proteins in its VGTs and proteomes (Huang and Mackessy 2004; Mackessy et al. 2020). This is the first and only record of a non-elapid snake genus harboring PLA_2_-I as a venom protein and might indicate that a duplication of the endogenous PLA_2_-IB gene, followed by sequential mutations toward an IA-like structure, occurred exclusively in this genus within Colubridae. On the other hand, PLA_2_s from group II appear to have been recruited into the venom glands of two separate families of rear-fanged snakes. These enzymes are arranged in a well-characterized cluster that is conserved in various vertebrate lineages and are known to be dominant toxins in vipers, where the PLA_2_-IIA gene, evolutionarily derived from PLA_2_-IID, has undergone several duplication/loss events. However, a different type of PLA_2_-II, PLA_2_-IIE, was recruited to the venom arsenal of some species of rear-fanged snakes within the Pseudoboini tribe (Dipsadidae) and the genus *Dispholidus* (Fry et al. 2012; Junqueira-de-Azevedo et al. 2016; Pla et al. 2017). We hypothesize that the PLA_2_-IIE gene suffered at least one event of duplication and shortening of the C-terminal tail after the Pseudoboini diversification from other Dipsadidae and this gene was recruited to the venom gland during the radiation of the *Clelia-like* group. A parallel recruitment of the PLA_2_-IIE gene occurred in Colubridae, specifically in the genus *Dispholidus*. The order in which these events took place and whether or not they occurred similarly or simultaneously in both groups is still a matter of investigation.

Moreover, these independent recruitments of PLA_2_-IIE and structural changes parallel the better-known evolutionary trajectories of group PLA_2_-I in Elapidae and group PLA_2_-IIA in Viperidae. Examples of independent recruitment of similar genes to become toxins in different snake groups are now becoming frequent and seem to indicate a trend toward the selection of a few optimal scaffolds to exert toxic functions in snake venom (Campos et al. 2016; Barua and Mikheyev 2019; Bayona-Serrano et al. 2020). The recruitment of PLA_2_-IIE to the venom glands of Pseudoboini represents a prime example of this trend.

In summary, although PLA_2_s are widespread venom components of several venomous snakes, we suggest that PLA_2_s became part of the venoms of Caenophidian snakes on at least five occasions and their appearance is not likely to be a basal trait selected early upon the divergence of the group. The PLA_2_-IIE gene was recruited and restricted to venoms of the rear-fanged families Colubridae (at least on *D. typus*) and Dipsadidae (Pseudoboini tribe) in two independent events, mirroring the recruitment and expansion of the PLA_2_-IIA gene in the Viperidae family (a third event). The PLA_2_-I gene, on the other hand, was apparently selected independently in both in Elapidae (fourth event) and in *Trimorphodon*, a specific Colubridae genus (fifth event). Since PLA_2_s are associated with some of the major toxic phenotypes of snake venoms, causing a wide array of effects, including cytotoxicity, myotoxicity, neurotoxicity, and many others, it is not surprising that these proteins have been selected multiple times during snake evolution. Nevertheless, the reiterated recruitment of nonvenom PLA_2_ genes in different snake families indicates that the intrinsic features of the PLA_2_ scaffold make it a valuable asset to effectively impose toxicity in different ecological contexts. Increased genomic sampling of rear-fanged snakes, along with increased functional and structural information from colubroid PLA_2_s, is still needed to shed light upon the complex evolutionary history of these toxins within snakes.

## Materials and Methods

### Collection and Storage of Samples

Specimens from eight genera and 19 species (IBAMA authorization 57585–1 and MAATE authorization MAE-DNB-CM-2019–0115) were collected during a series of field trips to different localities in Brazil and Ecuador. Venom samples were extracted using pilocarpin on sedated individuals as described in previous works (Mackessy et al. 2006). Four days after extraction, the venom glands and other tissues were surgically collected and stored in RNAlater at −80 °C.

### RNA Extraction and Analysis

Tissues were pulverized in a Precellys 24 homogenizer, and RNA was extracted with TRIzol (Invitrogen) following the modification of the method described by Chomczynski (1987) based on the use of guanidine isothiocyanate followed by phenolic extraction (Chomzynski 1987). Total RNA was quantified by the Quant-iTTM RiboGreen RNA reagent and kit (Invitrogen, Life Technologies Corp.). Quality control of the extracted RNA was then performed in an Agilent 2100 Bioanalyzer using an Agilent RNA 6000 Nano Kit to verify the integrity of total RNA through band discrimination corresponding to fractions 18S and 28S of total RNA. All procedures involving RNA were performed with RNase-free tubes and filter tips and using water treated with diethylpyrocarbonate (DEPC, Sigma). The general RNA integrity number (RIN) obtained for analyzed samples is available in supplementary figure S11, Supplementary Material online.

### cDNA Library Construction and Sequencing

Libraries were prepared for each individual sample. One microgram of total RNA was used with an Illumina TruSeq Stranded RNA HT kit consisting of TruSeq Stranded RNA HT/cDNA Synthesis PCR, TruSeq Stranded RNA HT/Adapter Plate Box, and TruSeq Stranded HT mRNA. Fragment size distributions were evaluated by microfluidic gel electrophoresis in a Bioanalyzer device (Agilent 2100) using an Agilent DNA 1000 Kit according to the manufacturer's protocol. Quantification of each library was then performed by real-time PCR using a KAPA SYBR FAST Universal qPCR Kit, according to the manufacturer's protocol, using the StepOnePlus Real-Time PCR System. Aliquots of each cDNA library were diluted to a concentration of 2 nM. Next, a pool of all samples, 5 *μ*l of each library, was prepared and the concentration of the pool was again determined by real-time PCR. The cDNA libraries were sequenced on an Illumina HiSeq 1500 System in Rapid Run mode using a paired-end flow cell for 300 cycles of 2*151 bp.

### Transcriptome Assembly and Annotation

To assemble the venom transcriptomes of the samples, we checked and removed cross-contamination using an in-house script (Hofmann et al. 2018) that compares sequences from other libraries within the sequencing pool and then trimmed the sequencing adaptors using TrimGalore (Krueger 2015). We merged our reads using PEAR software (Zhang et al. 2014) by taking advantage of the common overlap on the 3′ ends that characterizes paired-end short reads (Rokyta et al. 2012) and used those longer merged reads as an input for our assembly. We ran all the assemblies in a standardized way using five different assemblers with different k-mer values and assembly parameters (Trinity: k-mer 31; rnaSPADES: k-mer 31, 75, and 127; Extender: default, overlap 150, and seed size 2000; SeqMan Ngen: k-mer 21; and Bridger: k-mer 30) (Grabherr et al. 2011; Rokyta et al. 2012; Chang et al. 2015; Holding et al. 2018; Bushmanova et al. 2019). Then, we performed toxin annotation using ToxCodan (Nachtigall, Rautsaw, et al. 2021) against a curated data set of toxin sequences. Annotated toxin transcripts were manually reviewed and used to purge toxic-like contigs from the Trinity assembly of each individual. Then, both annotated toxin sequences and the remaining nontoxin Trinity contigs were combined to obtain a complete VGT of each individual, in which the toxin transcripts were curated. The coding sequences from nontoxin-purged contigs were predicted using CodAn with the full vertebrate model (Nachtigall, Kashiwabara, et al. 2021) and annotated by Blast searches against NCBI nr and PFAM following the ToxCodan pipeline available online (Nachtigall, Rautsaw, et al. 2021). The expression levels of each individual transcript were estimated using RSEM software (Li and Dewey 2011) after mapping the merged reads from each sample using Bowtie2 and were measured in transcripts per million (TPM) (Bankar et al. 2015).

### Proteomic Analyses

Analyses by reversed-phased nano chromatography coupled to tandem mass spectrometry analyses of the venoms from three species were performed by the Florida State University College of Medicine Translational laboratory and by the Laboratory of Toxinology (FIOCRUZ, Rio de Janeiro), as detailed in the supplementary methods, Supplementary material online. Protein identifications of the obtained spectra were performed using MASCOT (Matrix Science, London, UK; version 2.6.2) and X! Tandem (The GPM, thegpm.org, last accessed August 3, 2020; version X! Tandem Alanine [2017.2.1.4]) as the search engine. We considered a 99% and 95% threshold for protein and peptide identification, respectively. Custom-generated FASTA databases containing curated sequences of identified toxins for each specimen and translated protein sequences from the assembled transcriptome (Trinity contigs) for the species were used as a database for spectral identification, as detailed in the supplementary methods, Supplementary material online. To quantify the estimated abundance of each toxin class, we normalized the total spectra of all identified proteins using the Normalized Spectral Abundance Factor (NSAF) as implemented in Scaffold 5 (Zybailov et al. 2006).

### Venom Variation and Complexity within the Tribe

We transformed the expression data using the log-rate (center log-ratio [clr]) transformation method (Egozcue et al. 2003; Filzmoser et al. 2009) and applied the functions implemented in the *robCompositions* package (Templ et al. 2011) in the R environment. We used the *clr* transformation for visualization purposes, as it takes the simplex data into real space while retaining the individual identities of each toxin class. With these transformed values, we performed a principal component analysis (PCA) to evaluate the toxin compositions of the sampled Pseudoboini species. We used the *prcomp* function from the stats package in R version 4.1.0 (Team 2015). Then, we separated the poorly represented toxins (i.e., with average expressions of less than 1% of the total toxins) and grouped them into a category called “OtherToxins,” which was compared with the main toxins of the tribe. The graph was plotted using the *ggplot* package (Wickham 2016), and different colors were assigned for each analyzed species.

### Phylogenetic and Evolutionary Analyses of PLA_2_s in Snakes

We screened for PLA_2_-IIE-like contigs among four different snake families and seven additional tribes within Dipsadidae using an approach similar to that of Bayona-Serrano et al. 2020 (Bayona-Serrano et al. 2020). Briefly, we performed BlastN searches using de novo–assembled contigs from Trinity against the curated database of PLA_2_-IIE–like sequences obtained herein. The expression of each individual contig was calculated using RSEM (Li and Dewey 2011) by mapping the reads from each sample using Bowtie2. Expressions were estimated in TPM (Wagner et al. 2012, 2013). Afterward, PLA_2_-IIE–like contigs were identified and their expression values were added to obtain an approximate value for PLA_2_-IIE participation in each individual transcriptome. To better understand the phylogenetic history of the PLA_2_-IIE gene, we used the annotated PLA_2_-IIE sequences from our sampled individuals and combined them with the PLA_2_s from other publicly available vertebrates. The final nucleotide data set was then aligned through its corresponding translated amino acid sequences using the MUSCLE algorithm (Edgar 2004), with 20 iterations and default parameters in Geneious v.2020.0.5 software. Phylogenetic tree inference was then carried out using IQ-Tree2 (Minh et al. 2020) by combining the substitution model estimation with ModelFinder, a tree search with 1,000 replicates of ultrafast bootstrap and implementing the Shimodaira–Hasegawa approximate likelihood ratio test (SH-aLRT) and following the command recommended by the software developers. Moreover, we performed three additional tree searches using ultrafast bootstrap with 5,000 replicates, a nonparametric bootstrap with 1,000 replicates, both implemented in IQ-Tree2 (Minh et al. 2020) and a Bayesian approach in Mr. Bayes (Ronquist et al. 2012) using the nexus block available in the supplementary methods, Supplementary material online. Trees were visualized and edited using the iTol online platform (Letunic and Bork 2016). Orthology analyses were carried out for PLA_2_-IIE transcripts recovered from the tribe with OrthoFinder v2.4.0 to identify possible duplication events within the tribe (Emms and Kelly 2019). An inflation parameter of 0.5 was used.

### Enzymatic Assay for PLA_2_ Activity

Venom PLA_2_ activities were assayed in 96-well plates using 4-nitro-3- (octanoyloxy) benzoic acid (NOB) as substrate in 0.1 M Tris-HCl, pH 8, containing 0.01 M Ca2+ as reaction buffer for 30 min at 37 °C. The standard assay mixture contained 200 *μ*l of buffer, 20 *μ*l of substrate, and 20 *μ*l of venom (1 *µ*g/*µ*l) in a final volume of 240 *μ*l. After adding the venom, reactions were run in a SpectraMax 340 plate reader for 30 min at 37 °C, with the absorbance changes read at 425 nm. Venoms from nine species of Pseudoboini were tested. Crude venoms from the viper *B. jararacussu* and the CB subunit from the crotoxin of the rattlesnake, *Crotalus durissus*, were used as positive controls since they are recognized to have high PLA_2_ activities (Freitas-De-sousa et al. 2020; Montoni et al. 2020). Venoms from colubrid snakes, *Philodryas olfersii* and *Thamnodynastes chaquensis*, were used as negative controls, since the venoms of these genera were reported to have low or no PLA_2_ activity (Diaz et al. 2004; Ching et al. 2006; <bibref rid=“b14”>Correia et al. 2010, 2012; Zelanis et al. 2010; ). The obtained absorbance values were plotted for each sample, and the standard errors of the mean (SEM) were calculated for samples for which we had enough venom to run duplicate tests. To determine if there were significant differences among the groups, the nonparametric Kruskal–Wallis test was used. Multiple comparisons between the different groups were made through the nonparametric Wilcoxon test. All statistical tests were performed using R software version 4.1.0. Groups were considered significantly different if they had *P* < 0.05.

### Structural Analysis of the PLA_2_-IIE from Pseudoboini

To understand the structural differences between PLA_2_-IIEs from Pseudoboini and other snakes and the venom PLA_2_-IIA from vipers, we aligned the primary structures of the assembled PLA_2_-IIE to PLA_2_-IIA/IIE sequences from other publicly available snakes. We used the MUSCLE algorithm (Edgar 2004), with 20 iterations and default parameters in Geneious v.2020.0.5 software. Then, to see how those differences in primary structure might affect the 3D organization of each protein, we predicted the 3D structures of PLA_2_-IIE from Pseudoboini and other snake species using the RoseTTAFold method implemented in the Robetta protein structure prediction server (Hiranuma et al. 2021). Predicted protein structures were only considered for further analyses if they had a predicted local distance difference test (l-DDT) higher than 0.80. We downloaded the crystal structure of a catalytically active PLA_2_-IIA from *B. jararacussu* (UniProt code 1ZL7) and aligned it against our predicted models using the Matchmaker function available on ChimeraX 1.3 software. A fraction parameter of 1 was used to prioritize secondary structure over residue composition. The root-mean-square deviations of atomic positions (RMSD) of each alignment were used to estimate how well each of the models adjusted to the IIA structure.

### Phylotranscriptomic Analyses

First, we checked for putative sample contamination by assembling the mitochondrial sequences from each sample using MITGARD (v1.2) (Nachtigall, Grazziotin, et al. 2021) with the *Imantodes cenchoa* mitochondrial genome as reference (GenBank accession number EU728586.1). MITGARD is a tool that recovers the mitochondrial genome from RNA sequencing (RNA-seq) data by using a reference as bait to retrieve the mitochondrial reads and use it to assemble the mitogenome. We annotate the assembled mitogenomes using MitoZ (v2.4) (Meng et al. 2019). Then, we used the assembled and annotated mitochondrial sequences to compare with previously obtained mitochondrial sequences of Pseudoboini species to validate the species identity. We also used 15 annotated mitochondrial genes (i.e., two ribosomal and 13 protein coding) to infer a phylogenetic tree for each gene separately. To do this, we aligned their sequences using MAFFT (v7.310) (Katoh and Standley 2014), trimmed the alignments using trimAl (v1.2) (Capella-Gutiérrez et al. 2009) with the “-automated1” parameter, and built trees using IQ-TREE (v2.0.3) (Minh et al. 2020). Branches with Bootstrap values lower than or equal to 95 were removed from each mitochondrial gene tree using the Newick Utilities package (v1.6) (Junier and Zdobnov 2010), and the final consensus tree was generated using the coalescent approach implemented in Astral (v5.15.4) (Mirarab et al. 2014).

Then, we employed the software BUSCO (v5.2.2) (Manni et al. 2021), which infers measurements of genome and transcriptome completeness based on evolutionary informed expectations of gene content through the use of sets of lineage-specific sets benchmarking universal single-copy orthologs. We used the “aves_odb10” set (total of 8,338 genes in the BUSCO set) that represents the set with closer relationship to snakes among all other BUSCO sets and allowed the recovery of a higher number of nuclear loci to be used in the tree inference. We retrieved a total of 5,359 loci and filtered it to only keep loci containing at least 15 samples to avoid bias related to missing data, which resulted in a final set containing 2,161 loci. We aligned each locus separately in the final set using MAFFT (v7.310) (Katoh and Standley 2014) with the parameters “–auto” and “–adjustdirectionaccurately.” The alignments were cleaned using CIAlign (v1.0.14) (Tumescheit et al. 2022), with the following parameters “–remove_divergent –remove_divergent_minperc 0.80 –remove_insertions –crop_ends –remove_short.” The alignments were trimmed using trimAl (v1.2) (Capella-Gutiérrez et al. 2009) with the “-strictplus” parameter. The trimmed alignments were used to infer the phylogenetic trees for each locus using IQ-TREE (v2.0.3) (Minh et al. 2020). Then, branches with Bootstrap values lower than or equal to 95 were removed from each locus tree using the Newick Utilities package (v1.6) (Junier and Zdobnov 2010) and the final consensus tree was generated using the coalescent approach implemented in Astral (v5.15.4) (Mirarab et al. 2014).

## Supplementary Material

msad147_Supplementary_Data

## Data Availability

Raw transcriptomic data are available at NCBI's GenBank under Bioproject accession number PRJNA625548. Curated sequences (CDS) for all toxin transcripts generated in this work are available in supplementary table S1, Supplementary Material online, organized per species. Additional supplementary information (i.e., RAW proteomic data and multiple sequence alignments for phylogenetic analyses are available in a Figshare project [accessible at https://figshare.com/projects/Independent_recruitments_of_different_types_of_phospholipases_A2_to_the_venom_of_Caenophidian_snakes_the_rise_of_PLA2-IIE_within_Pseudoboini_Dipsadidae_/162772]).
