# Supplementary material for: Independent Recruitment of Different Types of Phospholipases A2 to the Venoms of Caenophidian Snakes: The Rise of PLA2-IIE within Pseudoboini (Dipsadidae)
Source: Mol Biol Evol. 2023 Jun 23;40(7):msad147. doi: 10.1093/molbev/msad147 (PMC10321490; doi:10.1093/molbev/msad147)
Supplement: msad147_Supplementary_Data [file msad147_supplementary_data.zip › SupplementaryMethods.pdf]

1. Proteomic analyses

1.1 Methodology used for samples processed at the Florida State University (FSU) School of Medicine Translational Laboratory

LC-MS/MS analyses for venom samples of the genus *Pseudoboa* were performed by the Florida State University (FSU) School of Medicine Translational Laboratory as previously described (Rokyta and Ala 2017; Ward and Rokyta 2018; Nystrom et al. 2019). The digested venom proteins were resuspended in 0.1% formic acid to reach a final concentration of 250 ng/µL. Highly purified recombinant proteins P31697 (Chaperone FimC protein), P31658 (Deglycase 1 protein), and P00811 (Beta-lactamase ampC) from *Escherichia coli* were added at concentrations of 2,500 fmol, 250 fmol, 25 fmol respectively by injection. The digested *E. coli* protein mixture was infused into the venom samples prior to LC-MS/MS injection. Approximately 2 µL of each sample was injected into an externally calibrated Thermo Q Exactive HF (high-resolution electrospray tandem mass spectrometer) in conjunction with the Dionex UltiMate3000 RSLC nano System to perform LC-MS/MS analysis. Samples were aspirated into a 50 µL loop and loaded onto the column (Thermo µPrecolumn 5 mm, with nanoViper tubing 30189µm i.d.×10 cm), using a flow rate of 300 nL/min for separation in the analytical column (Acclaim pepmap RSLC nanoViper of 75µm x 15 cm). A 60-minute linear gradient from 3% to 45% B was implemented using mobile phases A (99.9% H_2O (EMD Omni Solvent) and 0.1% formic acid) and B (99.9% ACN and 0.1% formic acid). The LC eluent was nanosprayed in a Q Exactive HF mass spectrometer (Thermo Scientific), which was operated in a data-dependent mode under the direct control of Thermo Excalibur 3.1.66 (Thermo Scientific). A data-dependent top-20 method was used for MS data acquisition, selecting the most abundant precursor ions from the survey scans (350–1700 *m/z*). Sequencing was performed under high energy collisional dissociation fragmentation using a target value of 10^5 ions determined with predictive automatic gain control. Full scans (350–1700 *m/z*) were performed at a resolution of 60,000 in profile mode, and MS2 were acquired in centroid mode at a resolution of 15,000, as described previously (Rokyta and Ward 2017; Ward et al. 2018; Ward et al. Rokyta 2018; Nystrom et al. 2019). Approximately 15

seconds was used as the dynamic exclusion window, and ions with a single charge, a charge of more than seven, or an unassigned charge were excluded. Measurements were made at room temperature and in triplicate for each sample to promote label-free quantification and to account for inter-sample variability.

The resulting raw files for all samples were searched using MASCOT (Matrix Science, London, UK; version 2.6.2) and X! Tandem [(The GPM, thegpm.org; version X! Tandem Alanine (2017.2.1.4)] as the search engine with custom generated FASTA databases containing curated sequences for each species. MASCOT search parameters used were as follows: maximum cleavage lost = 2, precursor mass tolerance = 10 ppm, fragment mass tolerance = 0.6 Da. Fixed modifications = Carbamidomethyl +57.021 Da (C). Dynamic modifications = Oxidation +15.995 Da (M). Protein and peptide identities were validated using Scaffold (version 4.9.0, Proteome Software Inc., Portland, OR, USA). Peptide and protein identities were accepted based on a 1.0% false discovery ratio (FDR) and a minimum identification of two unique peptides. The total number of spectrum counts was normalized using NSAF implemented in Scaffold 5 and used to estimate the relative abundance of the identified proteins.

1.2 Methodology used for the samples processed at the Laboratory of Toxinology (Proteomics Platform) of Oswaldo Cruz Foundation (FIOCRUZ), Rio de Janeiro, Brazil

Analyses were performed for venom samples from *Boiruna* and *Clelia* species. Peptide concentration from desalted tryptic digests was estimated by absorbance measurement at 280 nm (1.0 absorbance unit ≅ 1.0 µg/µL) on a NanoDrop 2000 spectrophotometer (Thermo Fischer Scientific) and normalized to 0.5 µg/µL with 1% formic acid in water as a diluent. Each sample was analyzed in three technical replicates by reversed-phase nanochromatography coupled to high-resolution nanoelectrospray ionization mass spectrometry. Chromatography was performed using an Easy-nLC 1200 system (Thermo Fischer Scientific). Samples (2 µL per run) were initially applied, at 2 µL/min of 0.1% (v/v) formic acid in water, to a 2-cm-long Thermo Acclaim trap column (75 µm inner diameter; 3 um matrix; part number 164535). Next, peptides were submitted to chromatographic

separation on a 38-cm-long column (75 µm i.d.), packed with ReproSil-Pur C18-AQ 120 Å 1.9 µm matrix (Dr. Maisch GmbH, Germany) directly onto a self-pack 10 um PicroFrit empty column (New Objective, USA). Fractionation was performed at 200 nL/min having 0.1% (v/v) formic acid in water and 0.1% (v/v) formic acid in 80% acetonitrile in water as mobile phases A and B, respectively. Elution was carried out from 2% to 30% B in 120 min; up to 45% B in 40 min; up to 100% B in 4 min, and maintained for 10 min more. The eluted peptides were introduced directly into a Q Exactive Plus Orbitrap instrument. Ionization was achieved by applying 1.9 kV to the source and setting the capillary temperature to 250 °C and the alternate current (radiofrequency) level of the S-lenses at 60 V. The complete MS1 scans (300 to 1,500 *m/z*) were acquired in the profile mode with one microscan at 70,000 resolution and an automatic gain control (AGC) target value of 1×10^6 with a maximum injection time of 100 ms. The 12 most intense precursor ions within the isolation window and offset of 2.0 and 0.5 *m/z*, respectively, were selected for HCD (higher-energy collision dissociation) fragmentation with collision energy normalized to 30 units. The MS2 spectra (200 to 2,000 *m/z*) were acquired in centroid mode with one microscan at 17,500 resolution and an AGC target value of 5×10^4 with a maximum injection time of 50 ms. Dynamic exclusion was set to 30 s, whereas peaks with unassigned charges or those with z = 1 were rejected.

The resulting raw files for all samples were searched using MASCOT (Matrix Science, London, UK; version 2.6.2) and X! Tandem [(The GPM, thegpm.org; version X! Tandem Alanine (2017.2.1.4)] as the search engine with custom generated FASTA databases containing curated sequences for each species. MASCOT search parameters were as follows: maximum cleavage lost = 2, precursor mass tolerance = 10 ppm, fragment mass tolerance = 0.02 Da. Fixed modifications = Carbamidomethyl +57.021 Da (C). Dynamic modifications = Carbamidomethyl +57.021 Da (D), Carbamidomethyl + 57.021 Da (E), Carbamidomethyl +57.021 Da (H), Carbamidomethyl +57.021 Da (K), Carbamyl +43.0058 (K), Carbamyl +43.0058 (N-term), Deamidation +0.9840 (NQ), and Oxidation +15.995 Da (M). Protein and peptide identities were validated using Scaffold (version 4.9.0, Proteome Software Inc., Portland, OR, USA). Peptide and protein identities were accepted based on a 1.0% false discovery ratio (FDR) and a minimum identification of two unique peptides. The total number of spectrum counts was normalized using NSAF

implemented in Scaffold 5 and used to estimate the relative abundance of the identified proteins.

2. Phylogenetic reconstruction of PLA_2s using a Bayesian approach.

The Bayesian analysis used to reconstruct the PLA_2 phylogeny, as depicted in supplementary figure S9, was carried out in Mr. Bayes (Ronquist et al. 2012) using the following NEXUS block on the PLA_2 alignment:

```
begin mrbayes;

set autoclose = no nowarn=no;

charset pos1 = 1-.\3;
charset pos2 = 2-.\3;
charset pos3 = 3-.\3;
partition by_codon = 3: pos1, pos2, pos3;
set partition = by_codon;

lset applyto = (all) nst=6 rates = invgamma;
unlink revmat=(all) shape=(all) pinvar=(all)statefreq=(all) tratio= (all);
prset ratepr=variable;
showmodel;

mcmc ngen=20000000 nchains=4 printfreq=50000 samplefreq=50000 diagnfreq=500000
checkpoint=yes checkfreq=50000 stopval = 0.01 stoprule = yes;
sump relburnin=yes burninfrac=0.25;
sumt relburnin=yes burninfrac=0.25 conformat=simple;

end;
```
