# Supplementary material for: Independent Recruitment of Different Types of Phospholipases A2 to the Venoms of Caenophidian Snakes: The Rise of PLA2-IIE within Pseudoboini (Dipsadidae)
Source: Mol Biol Evol. 2023 Jun 23;40(7):msad147. doi: 10.1093/molbev/msad147 (PMC10321490; doi:10.1093/molbev/msad147)
Supplement: msad147_Supplementary_Data [file msad147_supplementary_data.zip › SupplementaryFigures_S1_S11.pdf]

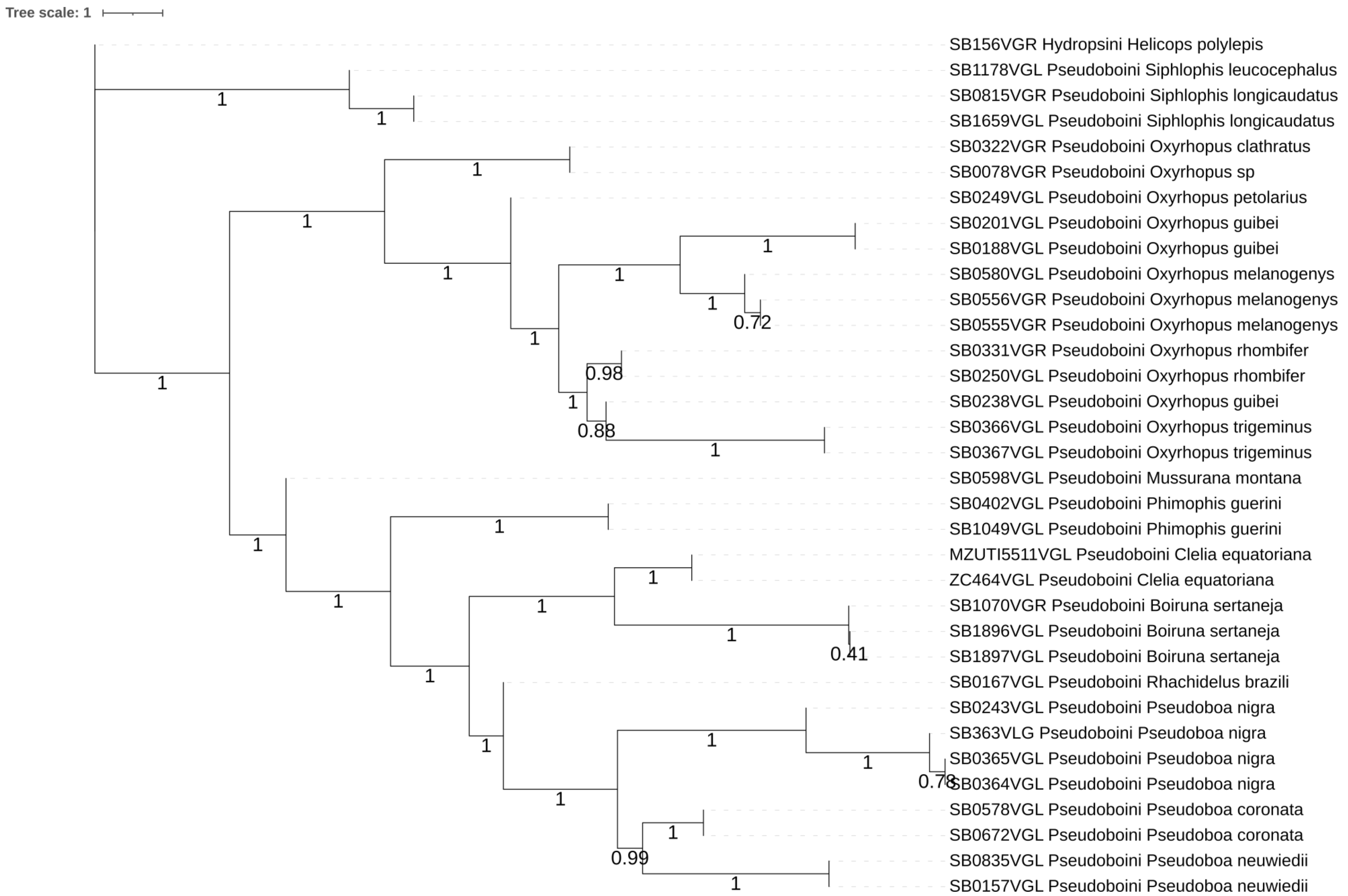

Supplementary figure 1. Maximum likelihood tree derived from conserved loci identified with BUSCO as described in the Materials and methods section. Bootstrap support values are shown for each node.

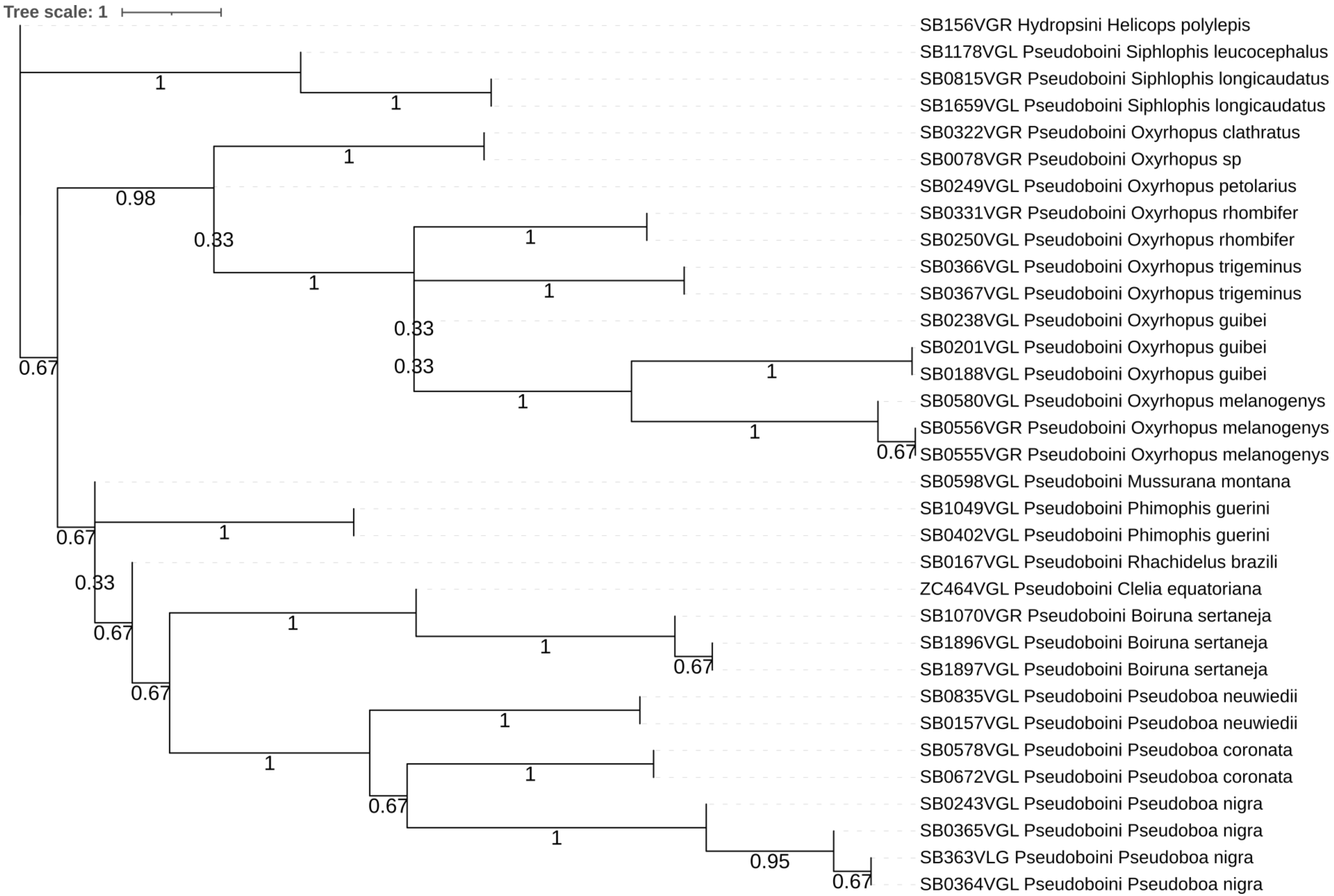

Supplementary figure 2. Maximum likelihood tree derived from recovered mitochondrial genomes assembled with MITGARD as described in the Materials and methods section. Bootstrap support values are shown for each node.

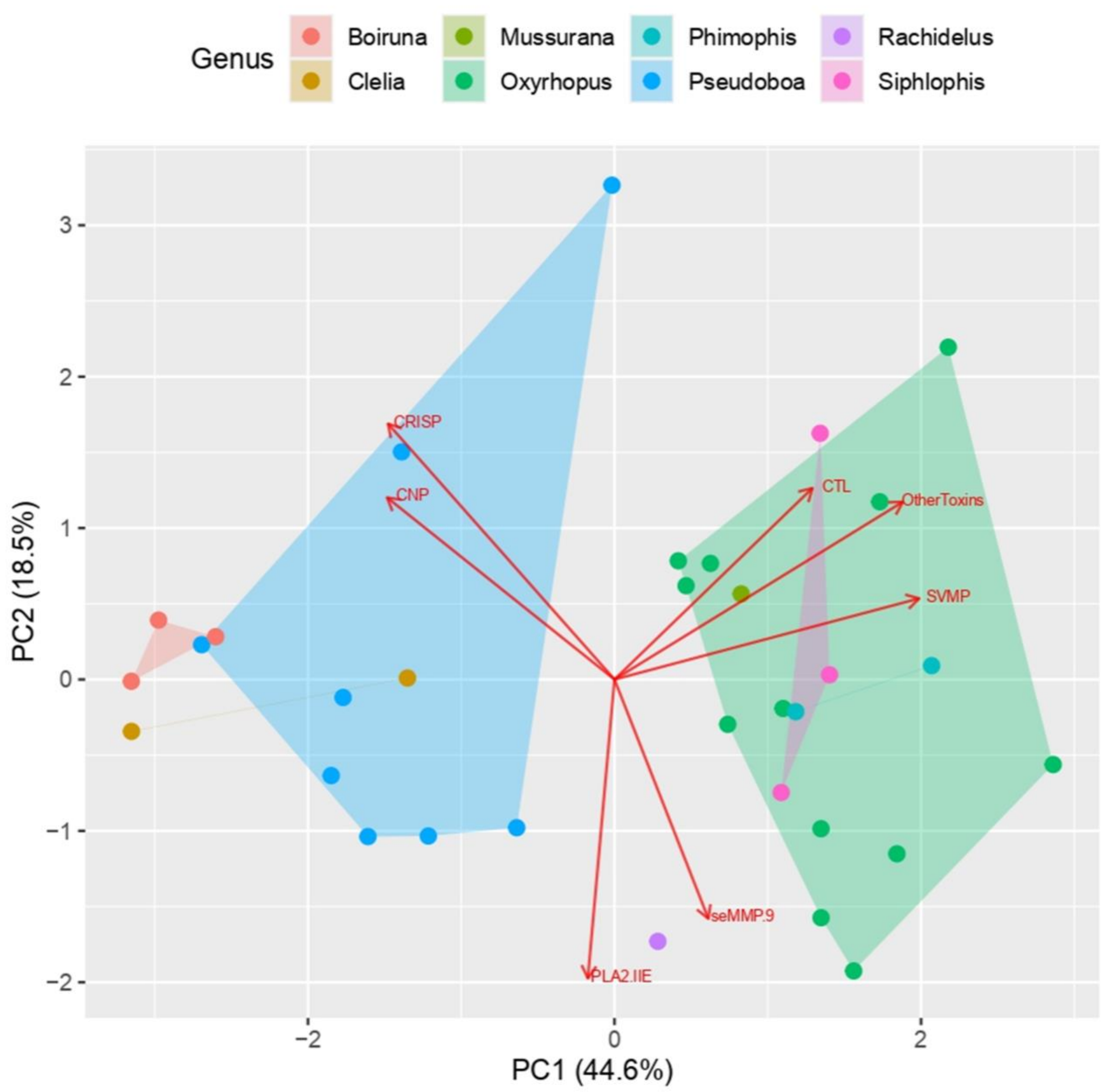

Supplementary figure 3. Principal Components Analysis (PCA) performed across the analyzed Pseudoboini species. Each genus is noted by a different color. Colored polygons indicate the observed intrageneric variation. The contribution of major toxins to venom variation is noted by red arrows. Species from *Pseudoboa*, *Clelia* and *Boiruna*, which posses the higher proportions of PLA2-IIE cluster to the left, separating from the rest of the tribe.

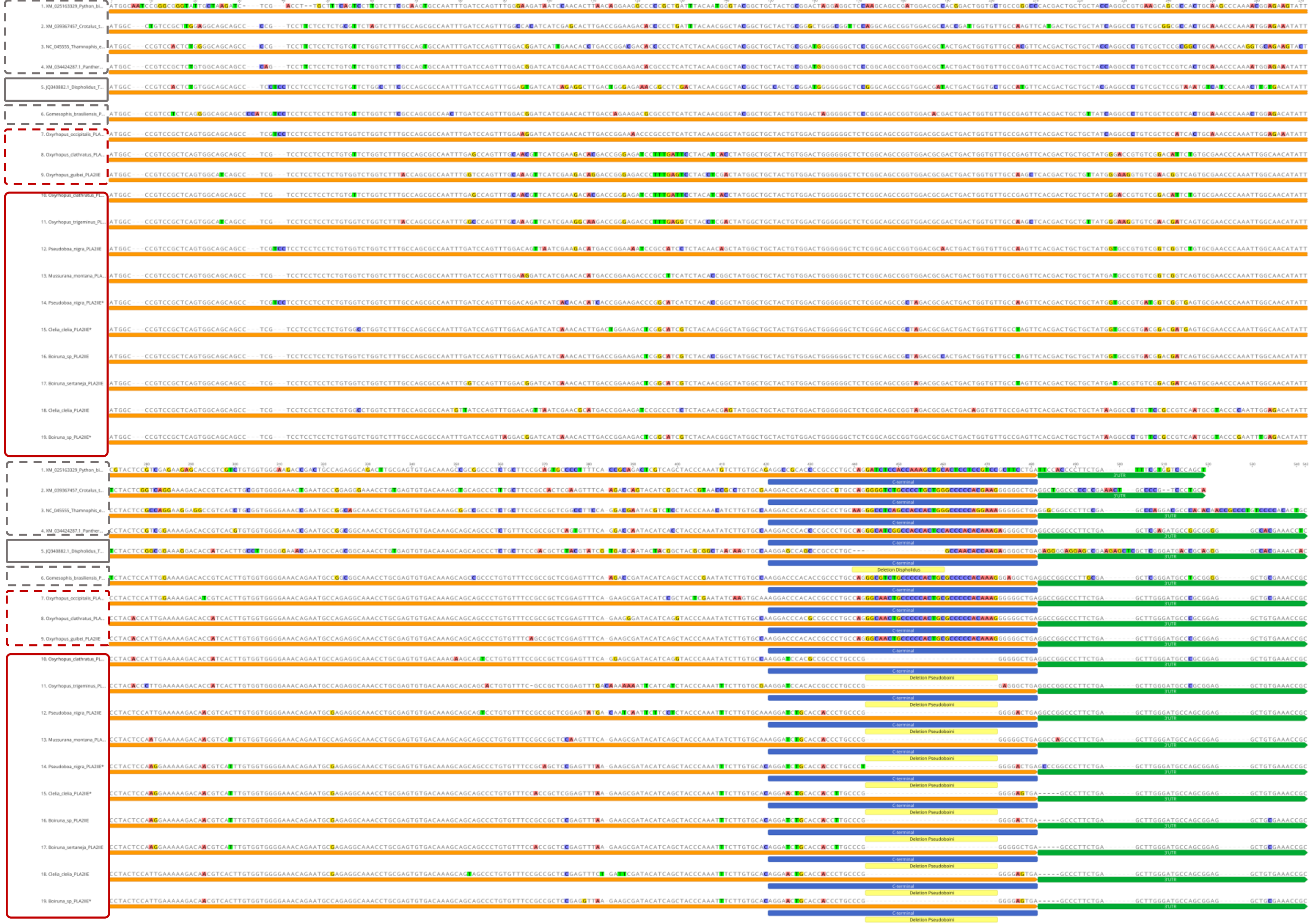

Supplementary figure 4. Multiple sequence alignment of PLA2-IIE contigs assembled herein and from previous works. Sequences obtained from external sources have their accession number in their ID. Structural features are noted by colored bars below each sequence as follows: The coding sequence is represented by an orange bar, the C-terminal portion of the protein is indicated by a blue bar, the 3'UTR is represented by a green bar. The presence of the C-terminal deletion is noted by a yellow bar. Sequence IDs from Pseudoboini species are marked with red boxes, all others are marked by grey boxes. Open boxes indicate sequences lacking the C-terminal deletion, while closed boxes indicate sequences harboring it. Within Pseudoboini we found both types of sequences, with the longer transcript being only found in the genus *Oxyrhopus*. Note the shorter deletion in *Dispholidus typus* PLA2-IIE when compared to the one observed in Pseudoboini species.

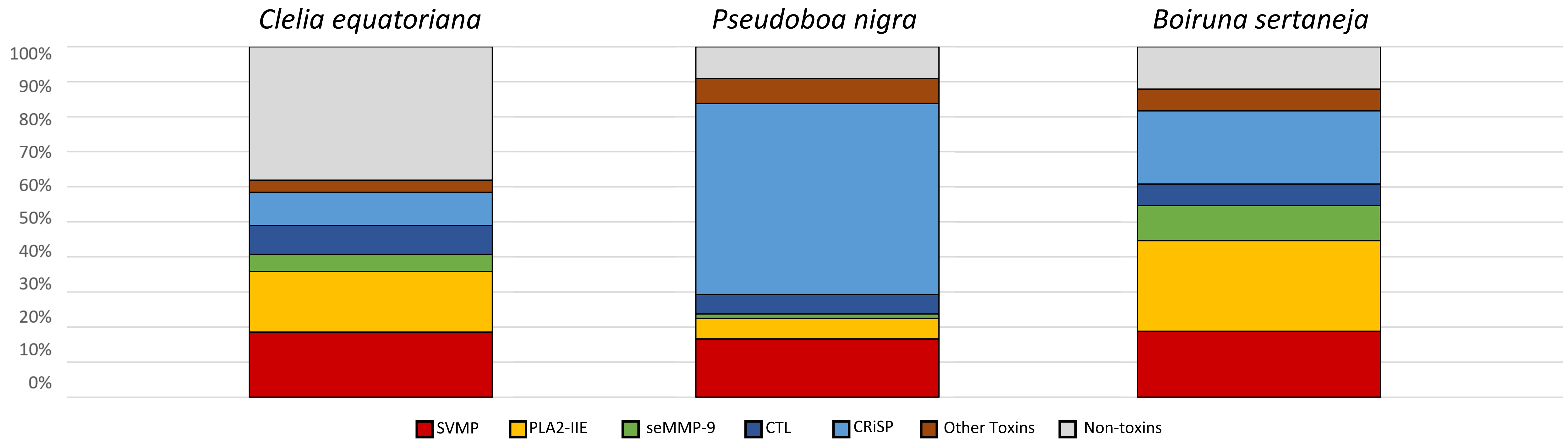

Supplementary figure 5. Proteomic estimated abundance of venom proteins. Total spectrum counts were normalized using NSAF and expressed in stacked graphs to compare the proportion of each toxin class among identified proteins. Venoms of the three species showed similar proportions of SVMPs, CTLs and minor toxins. *Clelia* and *Boiruna* showed higher proportions of PLA_2 while *Pseudoboa* showed a clear dominance of CRiSPs.

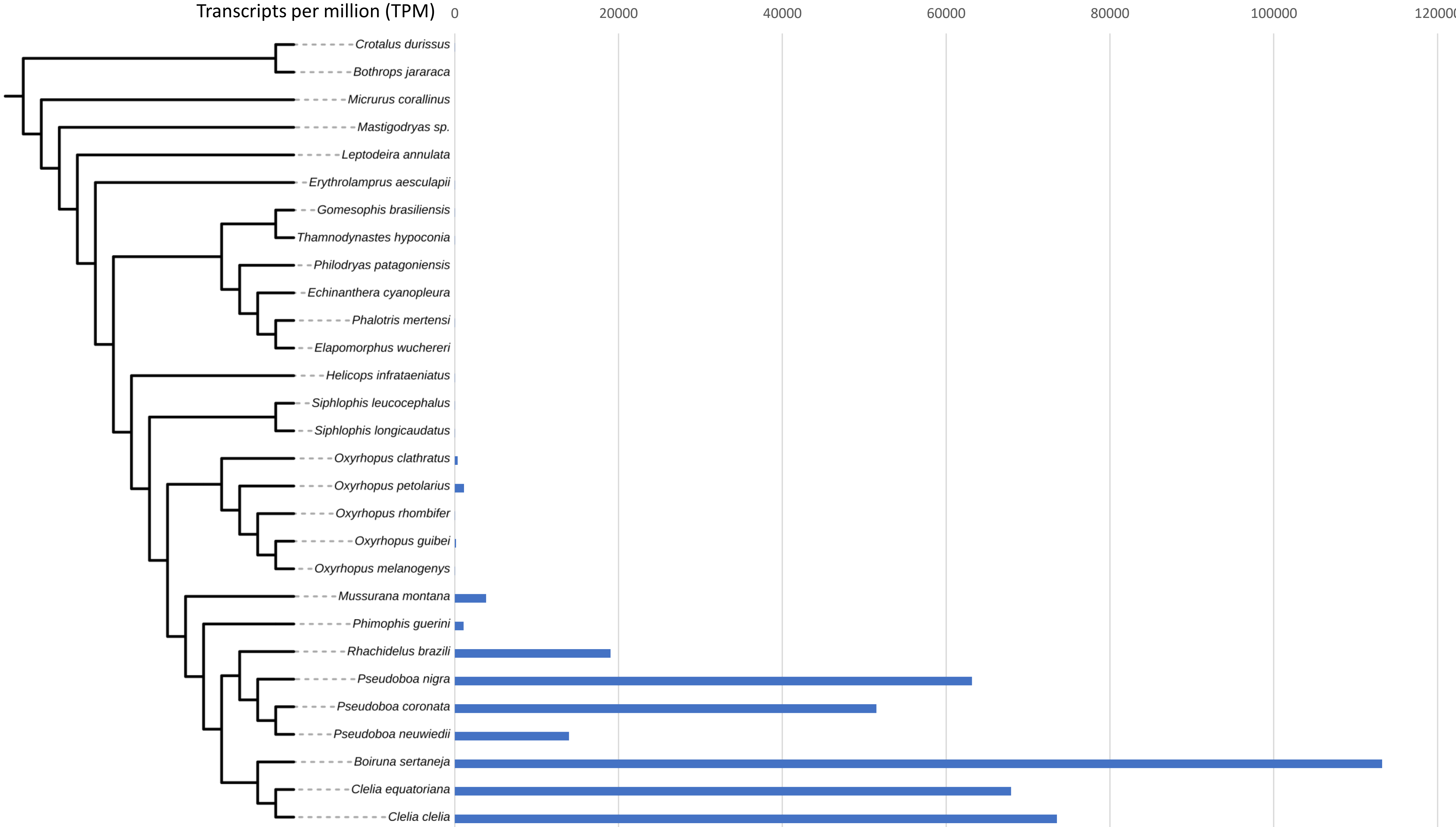

Supplementary figure 6. Occurrence of PLA2-IIE transcripts across snake phylogeny. Among screened species, elevated expression levels (shown in TPM) were only detected within the Pseudoboini tribe, specially within the genera *Mussurana, Rhachidelus, Pseudoboa, Clelia* and *Boiruna*, all belonging to the *Clelia-like* group.

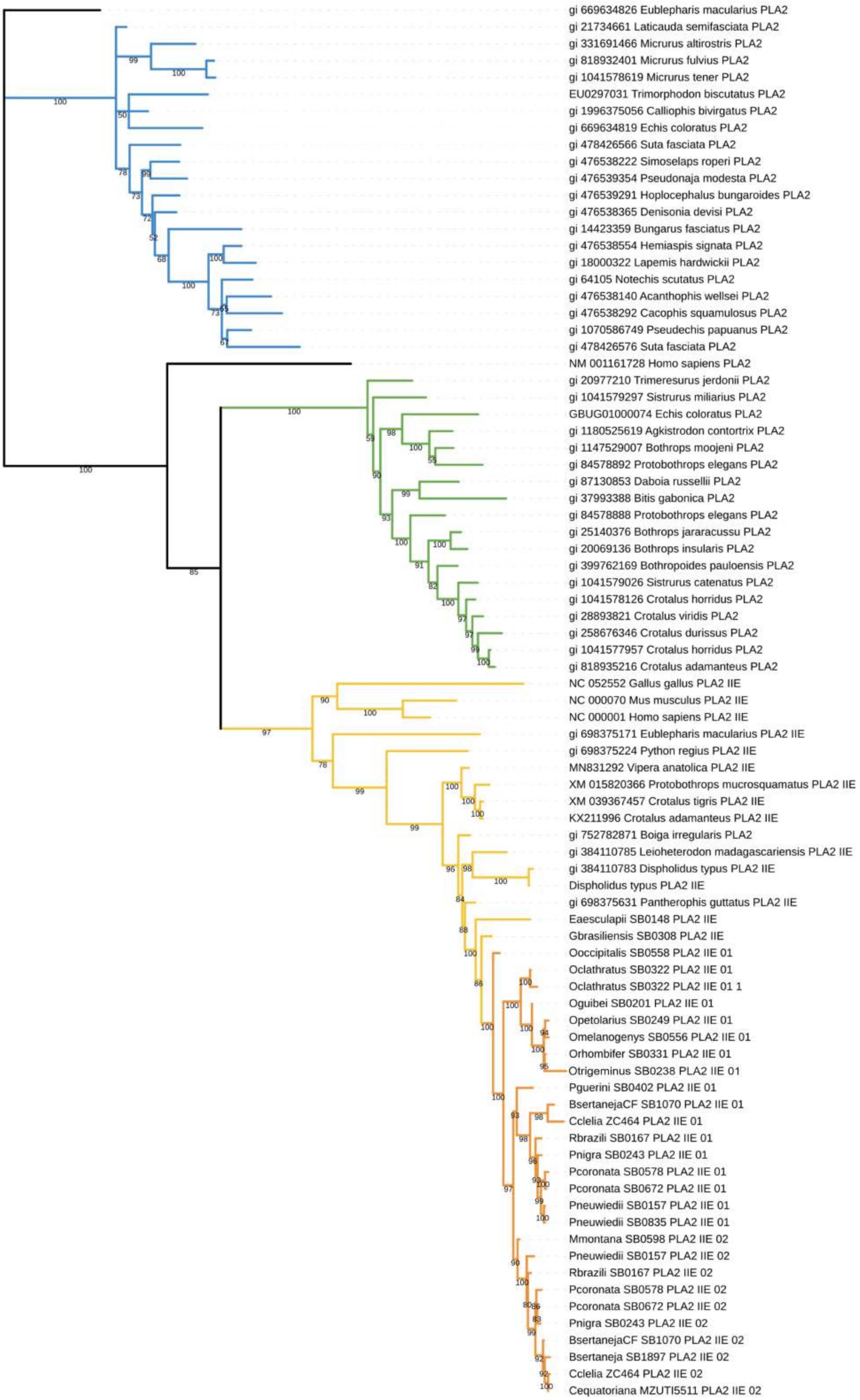

Supplementary figure 7. Maximum likelihood tree with 1000 ultrafast bootstrap replicates derived from PLA2-IIE sequences from Pseudoboini assembled herein and PLA2s from other snake groups as described in the Materials and methods section. Sequences obtained from external sources have their accession number in their label. Bootstrap support values are shown for each node and only node with a bootstrap value ≥50 were retained. PLA2s from group I are highlighted with blue branches. PLA2s from group IIA are highlighted with green branches. PLA2s from group IIE are highlighted with yellow branches, except for those belonging to the Pseudoboini tribe, which are distinguished by having orange branches.

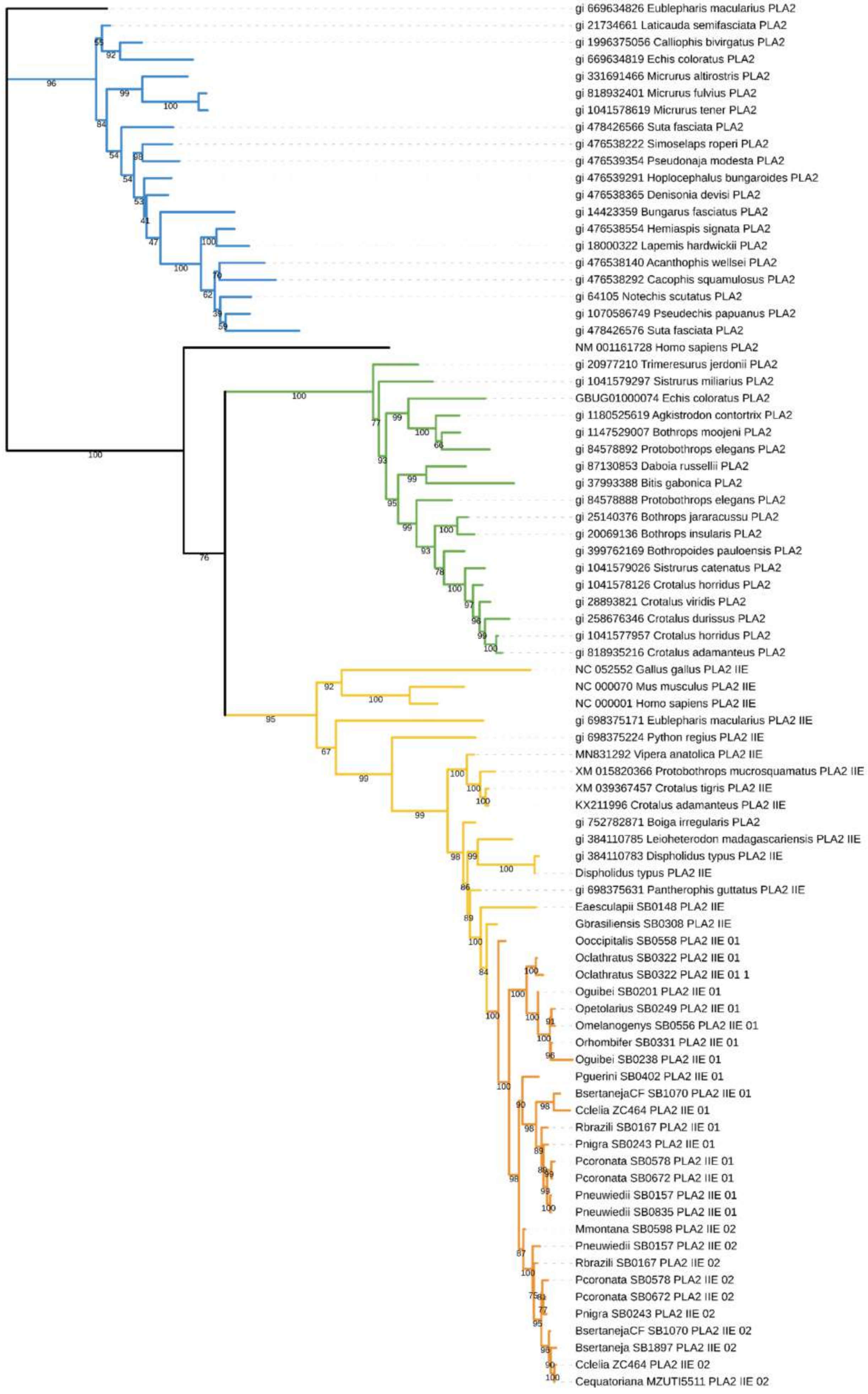

Supplementary figure 8. Maximum likelihood tree with 5000 ultrafast bootstrap replicates derived from PLA2-IIE sequences from Pseudoboini assembled herein and PLA2s from other snake groups as described in the Materials and methods section. Sequences obtained from external sources have their accession number in their label. Bootstrap support values are shown for each node. PLA2s from group I are highlighted with blue branches. PLA2s from group IIA are highlighted with green branches. PLA2s from group IIE are highlighted with yellow branches, except for those belonging to the Pseudoboini tribe, which are distinguished by having orange branches.

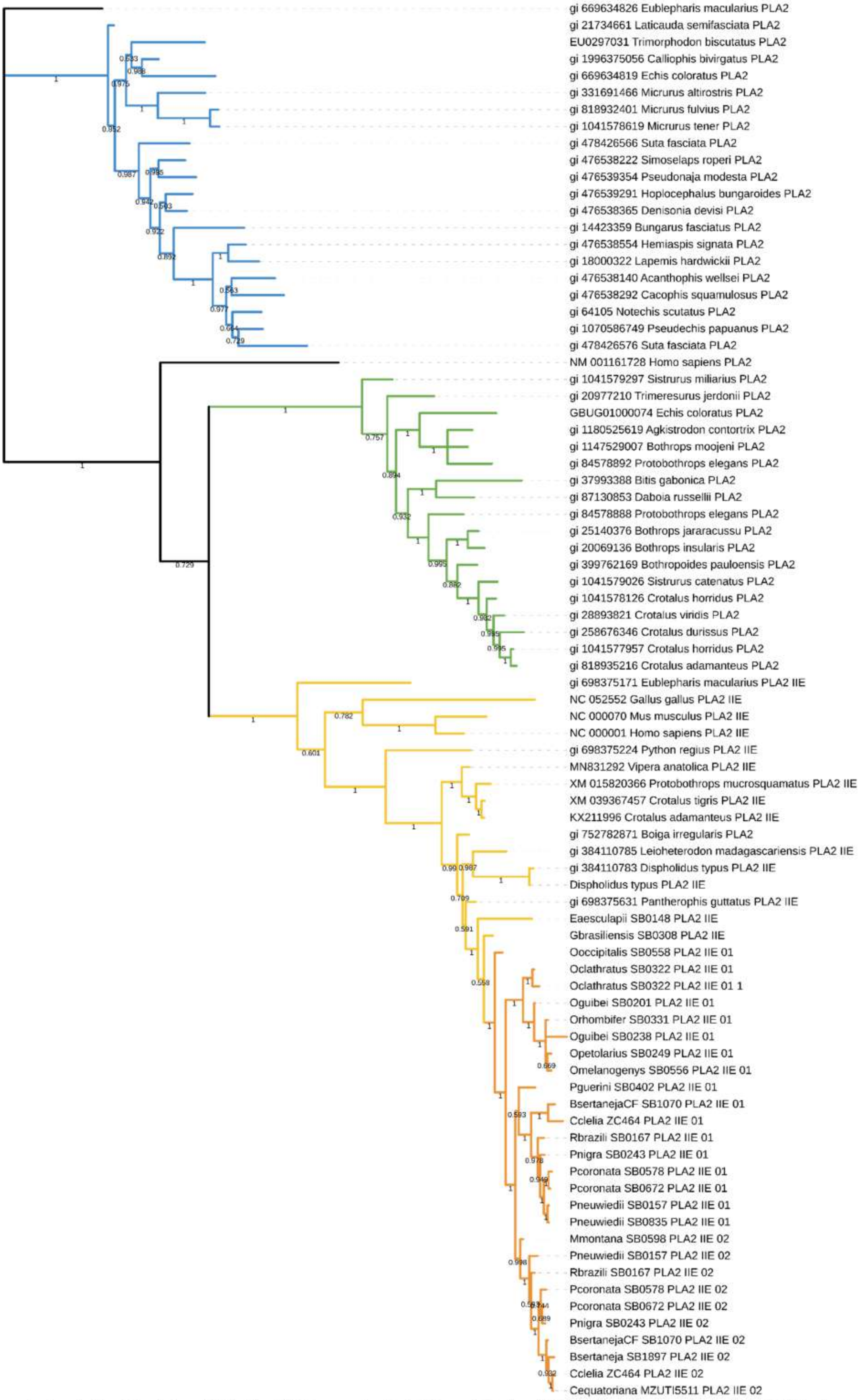

Supplementary figure 9. Bayesian tree with derived from PLA2-IIE sequences from Pseudoboini assembled herein and PLA2s from other snake groups as described in the Materials and methods section. Sequences obtained from external sources have their accession number in their label. Support values are shown for each node. PLA2s from group I are highlighted with blue branches. PLA2s from group IIA are highlighted with green branches. PLA2s from group IIE are highlighted with yellow branches, except for those belonging to the Pseudoboini tribe, which are distinguished by having orange branches.

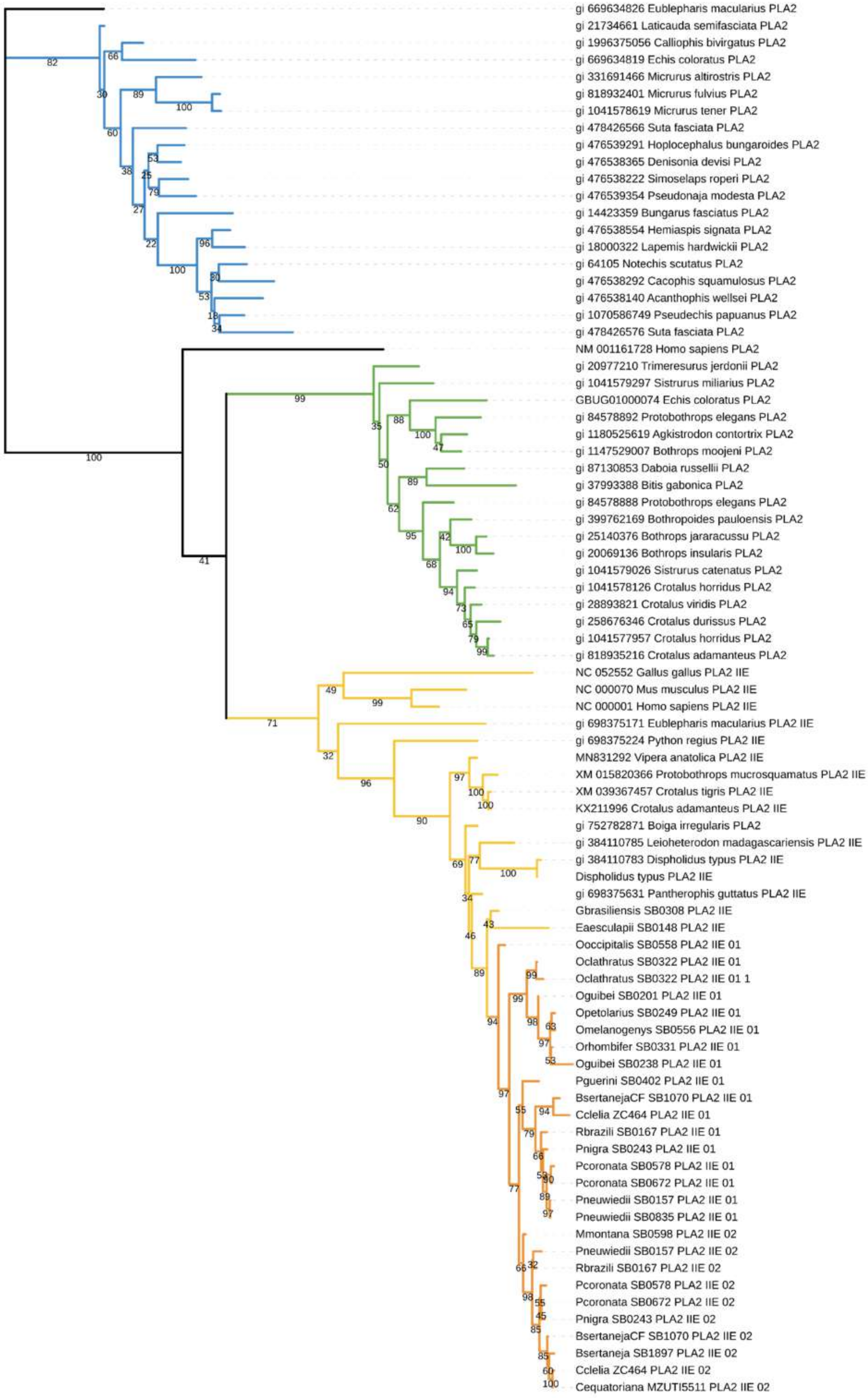

Supplementary figure 10. Maximum likelihood tree with 1000 non-parametric bootstrap replicates derived from PLA2-IIE sequences from Pseudoboini assembled herein and PLA2s from other snake groups as described in the Materials and methods section. Sequences obtained from external sources have their accession number in their label. Bootstrap support values are shown for each node. PLA2s from group I are highlighted with blue branches. PLA2s from group IIA are highlighted with green branches. PLA2s from group IIE are highlighted with yellow branches, except for those belonging to the Pseudoboini tribe, which are distinguished by having orange branches.

Sample	Species	RIN
SB0157VGL	*P. neuwiedii*	7.3
SB0167VGL	*R. brazili*	8.2
SB0188VGL	*O. guibei*	7.3
SB0201VGL	*O. guibei*	7.5
SB0238VGL	*O. trigeminus*	7.3
SB0243VGL	*P. nigra*	7.5
SB0249VGL	*O. petolarius*	7.6
SB0250VGL	*O. rhombifer*	6.8
SB0322VGR	*O. clathratus*	7.3
SB0363VGL	*P. nigra*	6.9
SB0364VGL	*P. nigra*	7.1
SB0365VGL	*P. nigra*	7.4
SB0366VGL	*O. trigeminus*	6.8
SB0367VGL	*O. trigeminus*	7.6
SB0402VGL	*P. guerini*	6.8
MZUTI_5809VGL	*C. clelia*	8.6
SB0555VGR	*O. melnogenys*	6.8
SB0672VGL	*P. coronata*	6.4
SB0815VGL	*S. longicaudatus*	6.4
SB0835VGL	*P. neuwiedii*	5.7
SB1049VGL	*P. guerini*	6.5
SB1070VGR	*B. sertaneja*	6.7
SB1178VGL	*S. leucocephalus*	6.2
SB1659VGL	*S. longicaudatus*	5.5
SB1896VGL	*B. sertaneja*	6.6
SB1897VGL	*B. sertaneja*	6.7
MZUTI_5511VGL	*C. equatoriana*	4.9

Supplementary figure 11. General profile of RIN values for analyzed samples. Most samples were between 6 and 8. For samples with lower RINs, we sequenced and assembled the transcriptomes, assessed their completeness with BUSCO searches and compared to that of other samples with better RIN values (data not shown). In all cases, the completeness of assembled transcriptomes was similar, so we kept those samples in subsequent analyses.
